# An interdisciplinary rehabilitation program for adults with dementia—A randomized controlled pilot trial evaluating social participation, loneliness and mental health

**DOI:** 10.1371/journal.pone.0345518

**Published:** 2026-03-24

**Authors:** Josefine Lampinen, Håkan Littbrand, Ingeborg Nilsson, Annika Toots, Yngve Gustafson, Jerry Öhlin, Henrik Holmberg, Birgitta Olofsson, Anita Ericsson, Mia Conradsson

**Affiliations:** 1 Department of Community Medicine and Rehabilitation, Umeå University, Umeå, Sweden; 2 Department of Public Health and Clinical Medicine, Sustainable Health, Umeå University, Umeå, Sweden; 3 Department of Epidemiology and Global Health, Umeå University, Sweden; 4 Department of Nursing, Umeå University, Umeå, Sweden; 5 Department of Diagnostics and Intervention, Orthopedics, Umeå University, Umeå, Sweden; 6 Department of Community Medicine and Rehabilitation, Geriatric Centre, Umeå University, Umeå, Sweden; University of the Witwatersrand Johannesburg, SOUTH AFRICA

## Abstract

**Background:**

To meet the complex needs of adults with dementia, a team-based, individualized rehabilitation approach may be required. This randomized controlled pilot trial evaluated the feasibility of a person-centred multidimensional interdisciplinary rehabilitation program for older adults with dementia, in terms of follow-up and response rates, and potential short- and long-term effects in adults with dementia on social participation, loneliness, and mental health.

**Methods:**

Participants (mean age (SD) 78.7 (±6.6) years), were randomized to an intervention group (n = 31) or usual care (n = 30). The rehabilitation program consisted of a 20-week rehabilitation period and two follow-ups after 5 and 14 months. An interdisciplinary team performed assessments and interventions based on the individual’s goals. Assessors blinded to group allocation performed structured assessments at baseline and after 5, 12, 24, and 36 months.

**Results:**

Initially, response rates in participants with dementia were high for all assessments in the areas of social participation, loneliness, and mental health. Response rates after 12 months decreased, particularly for cognitively demanding questions with multiple-choice options in the area of social participation. Overall, there were few statistically significant differences between the groups in the outcomes over 36 months, but some of the findings seemed potentially clinically meaningful in favor of the intervention group: increased frequency of active recreation and organized social activities outside the home, as reported by both participants with dementia and caregivers or staff; as well as experienced more frequent visits to family and friends; and short-term reduction in depressive symptoms.

**Conclusions:**

Assessments made of loneliness and mental health in this study over three years seemed feasible. It seemed cognitively demanding for participants with dementia to answer questions regarding social participation over time; therefore it seemed worthwhile to also ask informal caregivers or staff to avoid data loss. The positive findings noted during assessments and potential effects indicate that it is relevant to proceed further to an adequately powered RCT and conducted in additional geographical regions.

**Trial registration:**

ISRCTN – The UK’s Clinical Study Registry: http://www.isrctn.com/ISRCTN59155421

## Introduction

Dementia disorders are common among older adults and affects all aspects of life. Decline in cognitive functions and in the performance of meaningful occupations are some of the key characteristics of dementia [1,2]. The World Health Organization advocates rehabilitation be offered to adults with dementia and to their informal primary caregivers, intended to create favorable conditions for independent living and participation in society [3,4]. However, in contrast to other diseases engaging the central nervous system, e.g., multiple sclerosis [5] and stroke [6], rehabilitation programs are not routinely available for people with dementia in clinical settings [7–9]. Therefore, the Swedish government emphasizes in the updated dementia strategy (years 2025–2028) the importance of supporting measures that strengthen the conditions for rehabilitation to be initiated early in the course of the disease and followed up throughout its progression [10]. In addition to limited resources, reasons for the injustice in rehabilitation opportunities may include the challenge of managing the complex consequences related to dementia [11] as well as a lack of recognition that rehabilitation encompasses both the restoration and the preservation of functional abilities [12]. Furthermore, a negative attitude among rehabilitation staff and a lack of knowledge regarding the ability of adults with dementia to participate in rehabilitation may have an adverse impact [7,13–15].

Social participation—i.e., involvement in activities that provide interaction with others [16]—and performance in meaningful occupations are important in maintaining adults’ health and well-being [17,18]. However, among adults with dementia, the consequences of the disorder may limit the prerequisites for social participation. Such consequences include, e.g., reduced cognitive and functional ability; anxiety about participation in new activities and contexts [11]; lack of initiative and interest; and neuropsychiatric symptoms such as hallucination, agitation, and depression [19]. Social participation can also be negatively affected by others’ lack of understanding of dementia and of how to interact with affected persons [20]. Reduced social engagement may increase the risk of loneliness [21,22], which refers to a subjective feeling that occurs when there is a discrepancy between an individual’s desired and actual social relationships [23]. Reduced social engagement and experienced loneliness are associated with negative effects on health [24–27] including depression in older adults [28]. It is common for adults living with dementia to be offered antidepressant medication in cases of depressive symptoms, despite the literature indicating limited or no effect on depression in this group [29,30]. Alternative non-pharmacological interventions could be more beneficial [30,31], including rehabilitation with a focus on promoting social participation [32].

The heterogeneous and complex consequences of dementia suggest that person-centered multidimensional interdisciplinary rehabilitation efforts including many different health professionals are required [4,7]. In Sweden, however, only about half of the municipalities have dementia teams, and there is a shortage of many professions and competencies in these teams [33]. To date, scientific knowledge and clinical experience are limited concerning the effects of different rehabilitation models [34], and a need for more research into rehabilitation efforts for adults with dementia has been suggested [7,35]. Furthermore, it can be challenging to carry out rehabilitation programs in this group, including intervention evaluations, due to the complex symptoms in dementia. There is a need to investigate the feasibility of such programs, including the evaluation of follow-up and assessment rates of interview questions, especially questions with multiple choice options or questions that strain cognitive function, e.g., memory [36].

The Multidimensional Interdisciplinary Rehabilitation in Dementia (MIDRED) study is a randomised controlled pilot study evaluating the feasibility of a person-centred rehabilitation program for community-dwelling older adults with dementia, including education and support for informal primary caregivers [37–40]. The program was experienced as viable and beneficial by the older adults with dementia [38] and their informal caregivers [40], and there seemed to be some potentially clinically meaningful findings in the areas of gait, physical activity, neuropsychiatric symptoms, as well as being alive and continuing to live in the community [39]. The aim of the present study was to further evaluate the feasibility of the rehabilitation program in the MIDRED study, in terms of follow-up and response rates, and potential short- and long-term effects in adults with dementia on social participation, loneliness, and mental health.

## Method

### Setting and participants

The MIDRED study was conducted in Umeå, Sweden in 2016–2019. The study protocol was registered online at http://www.isrctn.com/ISRCTN59155421 before enrollment of the first participant. The MIDRED study was approved by the Regional Ethical Review Board of Umeå (Dnr 2015–292-31M; 2015–450–32 M). Adults with a dementia diagnosis, and their informal primary caregivers, were recruited from November 4th, 2015, to January 21st, 2016 at five local health centers in Umeå and the outpatient unit of the Geriatric Centre at the University Hospital in Umeå. Inclusion criteria were dementia diagnosis according to the International Statistical Classification of Diseases and Related Health Problems (ICD) 10th revision, 60 years and older, living in the community, the ability to rise from a chair with armrests with help from no more than one person, and the ability to hear and understand spoken Swedish sufficiently enough to participate in assessments. Additional inclusion criteria were a Mini Mental State Examination (MMSE) [41] score of 10 or higher, no initiated move into a nursing home, including respite care, expected survival of more than six months, and approval from the participants´ physician to participate in the study. Based on the information given about the study, staff at the six different units identified potential participants using lists of adults with dementia collected from medical records. If the individual accepted contact with the project staff, they received written information by post, and if they provided consent after a telephone contact by the researchers, a home visit was made. During this visit, the potential participant was assessed whether they met the inclusion criteria and were then given additional written and verbal information about the study. A maximum of two informal primary caregivers (spouse, child, or other relative or friend involved in the care of the person with dementia) of each participant with dementia were also invited to participate in the study after receiving oral and written information. All individuals in the study provided informed oral consent to participate, which was documented. Consistent with previous research, participants scoring ≥10 on the MMSE were considered capable of providing informed willingness to engage in an activity and of providing reliable feedback regarding their experiences with testing and training [42–45]. For the participants with dementia who were positive to participate in the study, their next of kin was also consulted and provided oral consent. The Regional Ethical Review Board of Umeå has approved this method of obtaining consent (see Supporting information File S1–S3).

### Interventions

The participants were randomized to a control group or an intervention group.

#### Control group.

Participants randomized to the control group received usual care; i.e., they continued their ordinary contact with the healthcare system according to their needs and planned follow-ups in outpatient settings. A local health center had primary responsibility for healthcare for all participants. In addition, if necessary in regard to dementia symptoms, they could also be referred to a specialist at the outpatient unit of the Geriatric Centre at the University Hospital in Umeå. There were no restrictions due to the study concerning the availability of rehabilitative efforts; e.g., by physiotherapists or occupational therapists.

#### Intervention group.

The participants who were randomized to the intervention group were offered participation in a rehabilitation program which included a 20-week rehabilitation period and two follow-up periods of four weeks each. They were conducted 5 and 14 months after the intervention period. The professions in the team included an assistant nurse, dental hygienist, dietician, geriatric medicine specialist, neuropsychologist, nurse, occupational therapist, pharmacist, physiotherapist, and social worker. The team practiced person-centered care [46], and all staff were experienced when it came to working with adults with dementia. During the initial four weeks of the rehabilitation period, team staff made assessments and identified every individual’s resources, problems, and needs within ten potential intervention areas for the person with dementia: ADL performance; social participation; functional capacity; cognitive function; physical activity; nutritional status; medical conditions, including oral health; neuropsychiatric symptoms of dementia; and pharmacological treatment. Intervention needs were defined based on the findings. Each participant with dementia and their informal primary caregiver(s), together with a maximum of two staff representatives, formulated individual rehabilitation goals, documented in a rehabilitation plan, and planned continuous follow-up meetings to evaluate goal fulfilment. All participants except one were active in establishing the rehabilitation plan [39]. Based on the goals, appropriate professionals formed a rehabilitation team for each participant and planned specific interventions. In weekly meetings including all team staff, the progress of the interventions and any new problems were discussed and evaluated. All ten professions delivered assessments and interventions to various extents [39].

Participants with dementia were offered interventions based on the individual´s goals by relevant professionals in the team, and physical exercise with social gatherings twice a week for 16 weeks. The interventions were conducted at the day rehabilitation unit at the Geriatric Centre, in the homes of participants with dementia and/or in the community. Examples of individually based interventions included the prescription of cognitive technical devices, support in engaging in household activities, introducing participants with dementia to a day-care centers and other activities in the community, guidance for achieving recommended levels of physical activity [47], social support regarding home services and finances, psychological support, review and correction of participants´ medication regimes, support to prevent malnutrition, interventions related to oral health, and medical controls. Physical exercise with social gatherings were offered in small groups twice a week at the day rehabilitation unit [42,48]. The exercise was individually tailored and supervised by physiotherapists. Participants were supported by team staff upon arriving and leaving the clinic. The assistant nurse arranged social gatherings during the coffee break after the exercise sessions, ensuring participants felt comfortable and welcomed.

For 5 and 14 months after the 20-week rehabilitation period, a follow-up of the rehabilitation were performed during 4 weeks, in which the team reassessed participants according to the same routine as the initial assessments. The interventions and activities that were initiated or proposed during the rehabilitation period were followed up and complementary interventions were offered if needed. For a more detailed description of the intervention program offered to participants with dementia, including assessments and interventions delivered, please see Hasselgren et al 2024 [39].

### Outcomes

#### Measurements.

During home visits, physiotherapists who were blinded to group allocation and earlier test results and trained with specific instructions for conducting testing conducted a structured interview and assessments at baseline and the consecutive follow-up assessments at 5, 12, 24, and 36 months**.** In the case of multiple-choice questions, the assessor read the alternatives and showed them in writing to make it easier for the participant with dementia. Additionally, oral questionnaires were administered to informal primary caregivers or, when necessary, care staff (either from social services at home or from a nursing home). To prevent the intervention from affecting the assessment of frequency of social participation, the 5-month follow-up assessment was scheduled at least 7 days after the end of the 20-week rehabilitation period. Data on participants’ medical history and current pharmacological treatment were collected by reviewing electronic medical records, in addition to the questionnaires. Dementia and depressive disorders were diagnosed according to the Diagnostic and Statistical Manual of Mental Disorders, fourth edition, text revision (DSM-IV,TR) [49], and the dementia diagnoses were verified using information from participants’ medical records, prescriptions, and assessments. At each follow-up, an experienced specialist in geriatric medicine (YG) reviewed all test protocols to assess whether participants had any serious medical conditions; e.g., severe depression or malnutrition. In those cases, their next of kin were informed and offered advice on how to get help. If applicable, a nurse or nursing staff were informed.

#### Follow-up rates.

Follow-up rates are presented as proportions of participants assessed, as well as structured interview questionnaires with caregivers or care staff completed through the study in the intervention and control groups, respectively. Reasons for missing data in participants with dementia assessments were retrieved from the Geriatric Depression Scale (GDS) and the Philadelphia Geriatric Center Morale Scale (PGCMS), noted as “cognitive impairment”, “cannot select alternative”, “declined”, or “other”.

#### Outcome measures.

Social participation for the participants with dementia was assessed via questions based on the Late-Life Function and Disability Instrument: Disability component (LLFDI-D) [50]. Based on the study design and target group, eight of nine questions were chosen from the LLFDI-D—e.g., frequency of keeping in touch and visiting family and friends, and participation in recreational and social activities in the community—and was complemented by the question “How often during a regular week do family or friends visit you in your own home” (visits by home care staff were not included). The participant with dementia and their informal caregiver were asked separately to estimate how often the participant with dementia participated in social activities during a regular week. The response alternatives were adapted from LLFDI-D, reducing the options from five to four, while also aligning the wording with the response choices in the loneliness question. The participant estimated the frequency as “often”, “sometimes”, “seldom”, or “never”. For the multiple-choice questions, the assessor read aloud the options while also displaying them in written form. The responses were then dichotomized to “often/sometimes” and “seldom/never”. The satisfaction with their frequency of social participation a regular week was estimated as “yes, satisfied”, “no, more often”, or “no, more seldom”. The answers were then dichotomized into “yes, satisfied” and “no, more often/more seldom”. The informal caregiver or care staff estimated the frequency in number of times, based on their knowledge of the participant’s social participation. The strategy to also ask caregivers or care staff to estimate social participation was implemented due to the anticipated cognitive challenge in these questions for the participants with dementia. Loneliness was measured with the question “Do you ever feel lonely?”. The participants with dementia rated their level of loneliness as “often”, “sometimes”, “seldom”, or “never” [26]. Following previous research, responses were dichotomized as lonely (“yes, often” and “yes, sometimes”) and not lonely (“no, seldom” and “no, never”) [26,51]. Depressive symptoms were assessed using the GDS 15-item version [43,52]. The scale has fifteen questions in a yes/no format. The score ranges from 0 to 15 and a score of 0–4 is considered within the normal range, 5–9 indicates mild depression, and a score of 10 or more indicates moderate to severe depression [52,53]. Psychological well-being were assessed using the PGCMS [54], including 17 questions with yes/no answers. The scores range from 0–17, where 0–9 indicates low psychological well-being, 10–12 indicates intermediate well-being, and 13–17 indicates high levels of psychological well-being [54,55].

#### Baseline measurements.

Educational levels were defined by the years of schooling they had received, either self-reported or reported by the next of kin. Global cognitive function was assessed using the MMSE (0–30) where a higher score indicates better function [41], and a score <18 indicates severe cognitive impairment [56]. Nutritional status according to Mini Nutritional Assessment (MNA, 0–30) [57] including body mass index (kg/m^2^), was assessed. An MNA score of less than 24 indicates a risk of malnutrition, and below 17 indicates the presence of malnutrition. Self-rated health was assessed using the first item of the 36-item Short-Form Health Survey, concerning general health [58]. The sense of safety was assessed with a single question; “Do you experience your life today as unsafe or safe?”, with five response alternatives (“pretty safe”, “very safe”, “hard to say”, “fairly unsafe”, and “very unsafe”). Dependence in ADL was assessed by questioning the informal caregiver or care staff using the Lawton and Brody scales [59,60]. The Lawton and Brody scale (14–61) contains six P-ADL domains rated on a scale ranging from 1 (independent) to 5 (total assistance) and eight I-ADL domains rated from 1 to 3–5. Usual gait speed with the habitual walking aid was assessed by a 2.4-meters timed test [61]. Vision and hearing were rated as impaired if they were unable to read a word printed in 5 mm capital letters with or without glasses or unable to hear a conversation held in a usual speaking voice from 1m with or without a hearing aid, respectively.

### Sample size and randomization

The original randomized controlled trial (RCT) aimed to recruit 179 participants with dementia, based on a power analysis of the proportion of people with dementia continuing to live in the community at the 24-month follow-up assessment (primary aim of the MIDRED study)[39]. Due to funding limitations, only the first group of participants out of the three planned groups was enrolled and randomized in the trial. Therefore, this study is reported as a randomized controlled pilot trial, evaluating the feasibility of the rehabilitation program [62]. The participants with dementia and their informal primary caregivers were randomized with a 1:1 allocation to a control group (usual care) or a person-centered multidimensional rehabilitation program for the person with dementia, including education and support for the informal primary caregiver. To prevent a random oblique distribution of participants´ characteristics between the groups, participants were stratified by type of dementia (Dementia with Lewy bodies or Parkinson´s disease with dementia, vascular dementia, Alzheimer´s disease, mixed Alzheimer´s disease and vascular dementia or unspecified dementia) and household living conditions (living alone or with a partner/child), and divided into 4*2 strata; i.e., four types of dementia and two living conditions. Within these strata, they were ranked according to their MMSE score. If the MMSE score was equal among participants, they were ranked according to age and sex (female sex first). Two persons not involved in the study performed the randomization with a block size of two from each stratum to balance intervention/control, using a die. Randomization was conducted following participant inclusion and completion of baseline assessments to minimize the risk of selection bias, thereby ensuring allocation concealment.

### Blinding

Assessment of outcome measures were performed by blinded assessors. If blinding was broken, the assessor was replaced according to a pre-planned schedule to preserve blindness. All follow-up assessments were carried out while maintaining blinding integrity [39].

### Statistical analyses

All analyses were based on the intention-to-treat principle, utilizing all available participant data based on their original group assignment, regardless of their attendance in the intervention. Baseline characteristics, outlined in Table 1 and selected a priori as potential confounders, were analyzed for differences between the intervention and control groups. These baseline differences were calculated using independent samples t-tests for continuous variables or chi-square tests for categorical variables. All between-group analyses were adjusted for age and sex, and any imbalances between groups at baseline (*p* < 0.05), according to our pre-established strategy; in this case, chronic lung disease, *p* = 0.04.

**Table 1:** Characteristics of the participants with dementia and baseline measures.

*Baseline characteristics*	Total, n = 60	Intervention, n = 31	Control, n = 29	*P-v*alue
*Age, years, mean ± SD*	78.9 ± 6.4	78.4 ± 6.0	79.5 ± 6.8	0.511
*Female, n (%)*	35 (58.3)	20 (64.5)	15 (51.7)	0.315
*Social service at home or remunerated help with P-ADL from informal caregiver n (%)*	22 (36.7)	12 (38.7)	10 (34.5)	0.734
*- Hours per week, mean ± SD*	2.6 ± 5.3	1.97 ± 3.15	3.33 ± 7.01	0.345
*Lives alone, n (%)*	21 (36.7)	10 (32.3)	11 (37.9)	0.464
*Education, year at school, mean ± SD*	11.0 ± 3.8	11.6 ± 3.7	10.3 ± 3.8	0.208
***Dementia type, n (%)***				
*Vascular*	11 (18.3)	5 (16.1)	6 (20.7)	
*Alzheimer’s disease (AD)*	29 (48.3)	16 (51.6)	13 (44.8)	
*Mixed (AD + vascular)*	11 (18.3)	5 (16.1)	6 (20.7)	
*Dementia with Lewy Bodies (DLB)***,** *Parkinson’s disease with dementia and mixed (DLB + vascular)*	9 (15.0)	5 (16.1)	4 (13.7)	0.537
***Diagnoses, n (%)***				
*Depressive disorders*	29 (48.3)	16 (51.6)	13 (44.8)	0.599
*Malignancy last 5 years*	25 (41.7)	13 (41.9)	12 (41.4)	0.965
*Chronic lung disease*	13 (23.3)	10 (32.3)	3 (10.3)	0.040
*Previous stroke*	11 (18.3)	5 (16.1)	6 (20.7)	0.648
*Heart failure*	12 (20.0)	6 (19.4)	6 (20.7)	0.897
*Angina pectoris*	10 (16.7)	4 (12.9)	6 (20.7)	0.419
*Osteoarthritis, lower extremity*	24 (40.0)	13 (41.9)	11 (37.9)	0.833
***Prescribed medication for regular use, n (%)***				
*Analgesics, N02A, N02B*	9 (15.0)	4 (12.9)	5 (17.2)	0.727
*Antidementia drugs:*				
*- Cholinesterase inhibitor, N06DA*	43 (71.7)	21 (67.7)	22 (75.9)	0.485
*- Memantine, N06DX01*	11 (18.3)	6 (19.4)	5 (17.2)	0.833
*Antidepressants, N06A*	19 (31.7)	9 (29.0)	10 (34.5)	0.650
*Antipsychotics, N05A*	4 (6.7)	4 (12.9)	0 (0)	0.113
*Benzodiazepines, N05BBA*	5 (8.3)	3 (9.7)	2 (6.9)	1.000
*Diuretics, C03*	14 (23.3)	8 (25.8)	6 (20.7)	0.640
*Number of medications mean* ± *SD (range)*	6.5 ± 3.1 (1-15)	6.4 ± 3.2 (1-14)	6.6 ± 3.1 (1-15)	0.836
***Assessments***				
*MMSE, 0–30, mean (SD), range*	21.0 (3.9), 9-30	20.8 (4.3), 9-30	21.1 (3.6), 15-28	0.823
*Vision; can read 5 mm capital letters, with or without glasses, n (%)*	59 (98.3)	31 (100)	28 (96.6)	0.483
*Hearing; can hear a conversation at normal voice level, with or without hearing aids, n (%)*	58 (96.7)	31 (100)	27 (93.1)	0.229
*MNA (0–30), mean ± SD (range)*	23.54 ± 2.9 (14-28)	23.44 ± 3.0 (17-28)	23.66 ± 2.9 (14-28)	0.502
*Self-reported health as good, very good or excellent, n (%)*	44 (73.3)	24 (77.4)	20 (69.0)	0.459
*Sense of safety (pretty safe, very safe), n (%)*	41 (68.3)	19 (61.3)	22 (75.9)	0.225
*L&B P-ADL score 6–30, mean ± SD (range)*	8.6 ± 3.4 (6-21)	8.8 ± 4.0 (6-17)	8.4 ± 2.7 (6-21)	0.621
*L&B I-ADL score, 8–31, mean ± SD (range)*	18.9 ± 5.8 (8-28)	18.7 ± 6.3 (10-28)	19.1 ± 5.4 (8-28)	0.787
*Usual gait velocity m/s, mean ± SD (range)*	0.71 ± 0.20 (0.19-1.09)	0.71 ± 0.23 (0.19-1.09)	0.71 ± 0.18 (0.24-1.09)	0.999

SD = standard deviation; MMSE = Mini Mental State Examination; MNA = Mini Nutritional Assessment; L&B P-ADL/ I-ADL = Lawton & Brody´s physical self-maintenance scale; P-ADL = personal activities of daily living; I-ADL = instrumental activities of daily living; m/s = meters/second. For all assessment scales, except L&B, higher scores indicate better function.

Longitudinal changes in caregivers’ estimation of the frequency of social participation and GDS-15, PGCMS answered by the participants with dementia, from baseline to 5, 12, 24, and 36 months were analyzed with Linear mixed-effect models. The models incorporated interaction terms for group and time-point and were adjusted for age, sex, and chronic lung disease as fixed effects and individuals as random effects. Baseline assessments were included in the outcome variable to avoid data loss. In the GDS-15 and PGCMS, missing values were imputed when at least two-thirds of the questions were answered. In GDS-15, the missing value was imputed with the mean of the answered questions, and the total score was then recalculated and rounded up to an integer. For PGCMS, missing answers were imputed with zero. For GDS-15, imputations were made in a total of 56 questionnaires throughout the study and in 60 questionnaires for the PGCMS, respectively. The number of questions with imputed answers ranged from 1–5 per questionnaire.

**Table 2:** Within- and between-group differences from baseline to the 36-month follow-up in frequency of social participation during a regular week among participants with dementia, estimated by informal caregivers or staff.

	Baseline value and within group difference*				Between group difference	
Measure	n	Intervention, mean (SE)	n	Control, mean (SE)	Mean (95% Confidence Interval)	p-value
***Keeping in touch with others through letters, telephone, or e-mail***						
*Baseline value*	*31*	*8.26 (1.67)*	*29*	*5.94 (1.86)*		
5 months	29	−2.46 (1.83)	29	−1.52 (1.85)	−0.94 (−6.08–4.20)	0.719
12 months	29	−3.56 (1.84)	28	−1.29 (1.87)	−2.27 (−7.44–2.90)	0.387
24 months	26	−3.13 (1.90)	24	−1.32 (1.97)	−1.81 (−7.20–3.58)	0.509
36 months	21	−1.00 (2.04)	21	1.06 (2.05)	−2.05 (−7.75–3.65)	0.479
***Visiting family and friends in their homes***						
*Baseline value*	*31*	*0.85 (0.28)*	*29*	*0.91 (0.31)*		
5 months	29	0.23 (0.34)	29	−0.10 (0.34)	0.33 (−0.62–1.28)	0.493
12 months	29	−0.22 (0.34)	28	0.31 (0.35)	−0.53 (−1.49–0.42)	0.274
24 months	26	−0.34 (0.35)	24	−0.20 (0.36)	−0.14 (−1.14–0.86)	0.782
36 months	21	−0.51 (0.38)	21	−0.69 (0.38)	0.17 (−0.88–1.23)	0.745
***Being visited by family and friends at home***						
*Baseline value*	*31*	*1.84 (0.43)*	*29*	*2.30 (0.48)*		
5 months	29	0.54 (0.49)	29	0.48 (0.49)	0.53 (−1.31–1.41)	0.939
12 months	29	0.05 (0.49)	28	0.62 (0.50)	−0.57 (−1.93–0.80)	0.415
24 months	26	−0.24 (0.50)	24	0.71 (0.52)	−0.95 (−2.37–0.47)	0.190
36 months	21	−0.35 (0.54)	21	0.44 (0.54)	−0.78 (−2.29–0.72)	0.306
***Taking part in active recreation***						
*Baseline value*	*31*	*2.64 (0.95)*	*29*	*4.93 (1.09)*		
5 months	29	2.13 (0.65)	29	1.70 (0.66)	0.44 (−1.39–2.27)	0.634
12 months	29	1.59 (0.65)	28	−0.08 (0.67)	1.66 (−0.18–3.50)	0.077
24 months	26	1.11 (0.68)	24	−0.49 (0.70)	1.60 (−0.32–3.53)	0.102
36 months	21	1.58 (0.73)	21	−1.26 (0.73)	2.84 (0.80–4.88)	0.007
***Taking part in organized social activities***						
*Baseline value*	*31*	*0.47 (0.20)*	*29*	*0.86 (0.22)*		
5 months	29	0.04 (0.24)	29	−0.14 (0.25)	0.18 (−0.50–0.86)	0.608
12 months	29	0.69 (0.24)	28	0.01 (0.25)	0.68 (0.00–1.37)	0.050
24 months	26	0.26 (0.25)	24	0.73 (0.26)	−0.47 (−1.18–0.24)	0.196
36 months	21	0.71 (0.27)	21	0.09 (0.27)	0.62 (−0.13–1.37)	0.105
***Going out with others to public places such as restaurants and movies***						
*Baseline value*	*31*	*0.23 (0.09)*	*29*	*0.20 (0.10)*		
5 months	29	0.08 (0.10)	29	−0.03 (0.10)	0.12 (−0.17–0.40)	0.423
12 months	29	0.01 (0.10)	28	−0.10 (0.11)	0.12 (−0.17–0.40)	0.428
24 months	26	−0.08 (0.11)	24	−0.06 (0.11)	−0.01 (−0.31–0.29)	0.935
36 months	21	0.05 (0.11)	21	−0.17 (0.11)	0.22 (−0.09–0.54)	0.168
***Taking care of local errands***						
*Baseline value*	*31*	*0.53 (0.16)*	*29*	*0.72 (0.18)*		
5 months	29	0.11 (0.20)	29	−0.14 (0.20)	0.24 (−0.31–0.80)	0.388
12 months	29	−0.27 (0.20)	28	−0.27 (0.20)	−0.004 (−0.56–0.55)	0.988
24 months	26	−0.34 (0.21)	24	−0.42 (0.21)	0.08 (−0.50–0.66)	0.789
36 months	21	−0.50 (0.22)	21	−0.36 (0.22)	−0.13 (−0.75–0.48)	0.672

* Change from the baseline value.

Within- and between-group differences were analyzed using linear mixed effect models adjusted for age, sex, and chronic lung disease.

**Table 3:** Within- and between-group differences from baseline to the 36-month follow-up in depressive symptoms and psychological well-being among participants with dementia.

	Baseline value and within group difference*				Between group difference	
Measure	n	Intervention, mean (SE)	n	Control, mean (SE)	Mean (95% Confidence Interval)	p-value
**Geriatric Depression Scale 15 item version (0–15)**						
*Baseline value*	*31*	*3.98 (0.53)*	*29*	*3.92 (0.60)*		
5 months	29	−0.44 (0.42)	29	0.38 (0.42)	−0.82 (−1.98-0.35)	0.169
12 months	29	−0.61 (0.42)	26	−0.03 (0.44)	−0.58 (−1.77-6.08)	0.335
24 months	25	−0.02 (0.44)	22	−0.13 (0.46)	0.11 (−1.14-1.37)	0.857
36 months	20	0.24 (0.47)	19	0.03 (0.49)	0.21 (−1.13-1.55)	0.756
**The Philadelphia Geriatric Center Morale Scale (0–17)**						
*Baseline value*	31	*11.97 (0.57)*	29	*12.15 (0.65)*		
5 months	29	0.04 (0.47)	29	−0.45 (0.47)	0.49 (−0.8-1.80)	0.461
12 months	29	−0.10 (0.47)	26	−0.26 (0.49)	0.16 (−1.1-1.50)	0.810
24 months	25	−0.33 (0.49)	22	0.66 (0.52)	−1.00 (−2.40-0.41)	0.163
6 months	20	−0.16 (0.53)	19	0.15 (0.54)	−0.31 (−1.80-1.19)	0.687

Within- and between-group differences were analyzed using linear mixed effect models adjusted for age, sex, and chronic lung disease. Geriatric Depression Scale 15-item version (0–15), higher scores indicate more depressive symptoms. The Philadelphia Geriatric Center Morale Scale, higher score indicates better psychological wellbeing.

Logistic regression was performed to assess differences between groups regarding experienced frequency of social participation (“often/sometimes” versus “seldom/never”), satisfaction with social participation (“satisfied” versus “no, more often/more seldom”), and extent of loneliness (“often/sometimes” versus “seldom/never”) at 5, 12, 24, and 36 months, separately, adjusted for age, sex, and chronic lung disease. All analyses were performed using SPSS software (version 28 for Windows; IBM Corporation, Armonk, NY, USA).

**Table 4:** Frequency of aspects of social participation experienced by participants with dementia as often/sometimes during a regular week, from baseline to 36-month follow-up.

Measure	n	Intervention, n (%)	n	Control, n (%)	OR (95% CI)	p-value
***Keeping in touch with others through letters, phone, or e-mail***						
*Baseline*	*31*	*25 (80.6)*	*29*	*23 (79.3)*		
5 months	29	22 (75.9)	29	23 (79.3)	0.604 (0.15-2.43)	0.477
12 months	28	21 (75.0)	26	22 (84.6)	0.554 (0.12-2.68)	0.463
24 months	22	18 (81.8)	21	18 (85.7)	0.643 (0.11-3.70)	0.621
36 months	18	14 (77.8)	18	14 (77.8)	1.001 (0.20-5.03)	0.999
***Visiting family and friends in their homes***						
*Baseline*	*31*	*19 (61.3)*	*29*	*25 (86.2)*		
5 months	29	16 (55.2)	29	13 (44.8)	4.190 (1.03-17.11)	0.046
12 months	28	13 (46.4)	26	14 (53.8)	1.191 (0.33-4.29)	0.789
24 months	22	12 (54.5)	21	16 (76.2)	0.575 (0.12-2.78)	0.492
36 months	18	9 (50.0)	18	11 (61.1)	1.482 (0.21-10.29)	0.690
***Being visited by family and friends at home***						
*Baseline*	31	19 (61.3)	29	22 (75.9)		
5 months	28	20 (71.4)	29	23 (79.3)	1.091 (0.27-4.35)	0.902
12 months	28	20 (71.4)	26	21 (80.8)	0.972 (0.20-4.67)	0.971
24 months	22	14 (63.6)	21	20 (95.2)	0.237 (0.02-2.73)	0.248
36 months	18	9 (50.0)	18	14 (77.8)	0.383 (0.08-1.95)	0.248
***Taking part in active recreation***						
*Baseline*	*31*	*17 (54.8)*	*29*	*24 (82.8)*		
5 months	29	20 (69.0)	29	24 (82.8)	0.738 (0.17-3.18)	0.683
12 months	28	19 (67.9)	26	20 (76.9)	0.905 (0.22-3.66)	0.889
24 months	22	18 (81.8)	21	15 (71.4)	2.159 (0.37-12.55)	0.392
36 months	17	13 (76.5)	17	12 (70.6)	3.299 (0.28-39.15)	0.344
***Taking part in organized social activities***						
*Baseline*	*31*	*9 (29.0)*	*29*	*9 (31.0)*		
5 months	29	9 (31.0)	29	8 (27.6)	1.048 (0.31-3.52)	0.940
12 months	27	16 (59.3)	26	9 (34.6)	2.254 (0.66-7.69)	0.194
24 months	22	9 (40.9)	21	9 (42.9)	0.857 (0.21-3.50)	0.830
36 months	17	9 (52.9)	17	5 (29.4)	2.941 (0.63-13.83)	0.172
***Going out with others to public places such as restaurants or movies***						
*Baseline*	*31*	*9 (29.0)*	*29*	*13 (44.8)*		
5 months	29	10 (34.5)	29	12 (41.4)	0.819 (0.22-3.00)	0.764
12 months	28	8 (28.6)	26	8 (30.8)	1.309 (0.33-5.17)	0.701
24 months	22	6 (27.3)	21	6 (28.6)	1.169 (0.23-6.00)	0.852
36 months	18	6 (33.3)	18	5 (27.8)	2.525 (0.43-14.94)	0.307
***Taking care of local errands***						
*Baseline*	*31*	*20 (64.5)*	*29*	*16 (55.2)*		
5 months	29	14 (48.3)	29	18 (62.1)	0.460 (0.13-1.59)	0.220
12 months	27	16 (59.3)	26	13 (50.0)	1.318 (0.38-4.52)	0.661
24 months	21	14 (66.7)	21	10 (47.6)	2.011 (0.51-7.98)	0.320
36 months	17	8 (47.1)	16	6 (37.5)	1.355 (0.30-6.05)	0.690

Odds Ratio (OR) was estimated using multivariable logistic regression analysis adjusted for age, sex, and chronic lung disease.

**Table 5:** Proportion of participants with dementia reporting themselves as satisfied with their experienced frequency of social participation during a regular week, as well as reporting feeling lonely often or sometimes over 36-month follow up.

	n	Intervention, n (%)	n	Control, n (%)	OR (95% CI)	p-value
***Keeping in touch with others through letters, phone, or e-mail***						
*Baseline*	*31*	*27 (87.1)*	*29*	*26 (89.7)*		
5 months	29	20 (69.0)	29	27 (93.1)	0.117 (0.01-1.00)	0.049
12 months	28	20 (71.4)	26	24 (92.3)	0.267 (0.04-1.61)	0.150
24 months	22	15 (68.2)	21	17 (81.0)	0.366 (0.06-2.32)	0.285
36 months	18	15 (83.3)	18	14 (77.8)	1.621 (0.21-12.29)	0.640
***Visiting friends and family in their homes***						
*Baseline*	*31*	*22 (71.0)*	*29*	*24 (82.8)*		
5 months	29	17 (58.6)	29	22 (75.9)	0.594 (0.15-2.39)	0.463
12 months	28	20 (71.4)	26	21 (80.8)	0.910 (0.21-3.91)	0.899
24 months	22	15 (68.2)	21	18 (85.7)	0.407 (0.08-2.05)	0.276
36 months	17	14 (82.4)	18	15 (83.3)	1.130 (0.16-8.01)	0.903
***Being visited by family and friends at home***						
*Baseline*	*31*	*20 (64.5)*	*29*	*24 (82.8)*		
5 months	27	17 (63.0)	29	23 (79.3)	0.741 (0.17-3.19)	0.687
12 months	28	18 (64.3)	26	21 (80.8)	0.721 (0.17-3.07)	0.658
24 months	22	13 (59.1)	21	19 (90.5)	0.161 (0.02-1.39)	0.097
36 months	17	12 (70.6)	18	13 (72.2)	1.378 (0.24-7.83)	0.717
***Taking part in active recreation***						
*Baseline*	*31*	*25 (80.6)*	*29*	*27 (93.1)*		
5 months	29	20 (69.0)	29	25 (86.2)	0.380 (0.09-1.55)	0.177
12 months	28	24 (85.7)	26	22 (84.6)	1.415 (0.26-7.69)	0.688
24 months	22	16 (72.7)	21	14 (66.7)	1.054 (0.25-4.54)	0.944
36 months	17	10 (58.8)	17	13 (76.5)	0.526 (0.11-2.53)	0.422
***Taking part in organized social activities***						
*Baseline*	*31*	*28 (90.3)*	*29*	*28 (96.6)*		
5 months	29	23 (79.3)	29	26 (89.7)	0.449 (0.09-2.25)	0.331
12 months	27	23 (85.2)	26	21 (80.8)	1.819 (0.26-12.60)	0.545
24 months	22	17 (77.3)	21	16 (76.2)	1.552 (0.30-7.96)	0.598
36 months	17	13 (76.5)	17	12 (70.6)	1.709 (0.30-9.90)	0.550
***Going out with others to public places such as restaurants or movies***						
*Baseline*	*30*	*25 (83.3)*	*29*	*27 (93.1)*		
5 months	29	24 (82.8)	29	23 (79.3)	1.874 (0.38-9.18)	0.438
12 months	28	24 (85.7)	26	22 (84.6)	1.197 (0.24-6.10)	0.829
24 months	22	18 (81.8)	21	17 (81.0)	2.780 (0.37-21.17)	0.324
36 months	18	16 (88.9)	18	15 (83.3)	1.442 (0.17-12.18)	0.737
***Taking care of local errands***						
*Baseline*	*31*	*29 (93.5)*	*29*	*26 (89.7)*		
5 months	29	26 (89.7)	29	25 (86.2)	1.245 (0.13-11.93)	0.849
12 months	27	23 (85.2)	26	24 (92.3)	0.441 (0.07-2.85)	0.390
24 months	21	17 (81.0)	21	18 (85.7)	0.852 (0.15-4.89)	0.858
36 months	17	16 (94.1)	16	15 (93.8)	1.119 (0.06-20.96)	0.940
**Feeling lonely often/sometimes**						
*Baseline*	*31*	*12 (38.7)*	*29*	*8 (27.6)*		
5 months	29	15 (51.7)	29	13 (44.8)	0.854 (0.24-3.09)	0.810
12 months	29	12 (41.4)	26	8 (30.8)	1.131 (0.23-5.56)	0.879
24 months	24	10 (41.7)	22	8 (36.4)	0.739 (0.14-4.01)	0.726
36 months	20	9 (45.0)	18	4 (22.2)	4.850 (0.65-36.49)	0.125

Odds Ratio (OR) was estimated using multivariable logistic regression analysis adjusted for age, sex, and chronic lung disease.

## Results

### Participants

**Fig 1:**
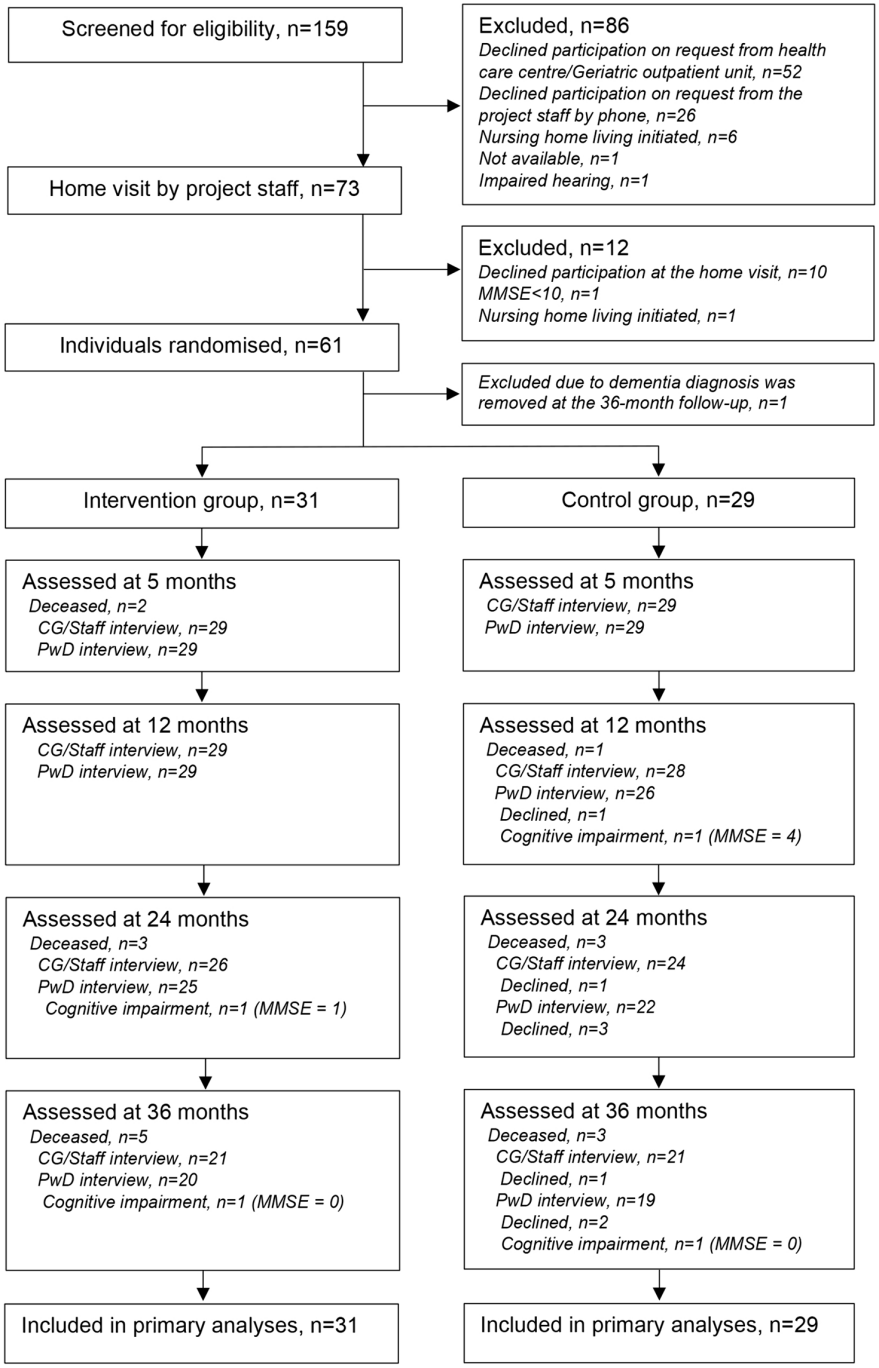
Flow of participants with dementia through the study. MMSE = Mini Mental State Examination (0-30), higher score indicates better cognitive function. PwD = Participant with Dementia; CG/Staff = Informal Caregiver and/or Staff. Informal Caregiver/Staff interview indicates the number of participants with dementia where data was collected via interviews with caregivers/staff. Reason for missing data of PwD interview has been retrieved from assessment of the Geriatric Depression Scale 15-item version and the Philadelphia Geriatric Center Morale Scale.

Initially, 159 individuals were screened, and of those, 61 individuals with dementia and 67 caregivers fulfilled the eligibility criteria (Fig 1). One participant with dementia and the associated informal primary caregiver were excluded from the statistical analysis as the Alzheimer´s disease diagnosis was, according to the medical records, removed at the 36-month follow-up. Thus, 31 participants with dementia in the intervention group and 29 in the control group were included in the final analysis. There was no significant difference in sex (p = 0.554) between participants included in the study and those who declined, however those who declined were older (mean age of 78.9 years compared to 82.2 years, p = 0.007).

Baseline characteristics according to group allocation are presented in Table 1. In total, the mean (SD) age was 78.9 (6.4) years, 35 individuals (58.3%) were female, the mean (SD) MMSE score was 21.0 (3.9), and six individuals had severe cognitive impairment (MMSE score <18). MMSE scores (mean [SD]) for the consecutive follow-ups at 5, 12, 24, and 36 months were 20.6 (4.9), 19.1 (6.0), 19.1 (6.2), and 17.4 (7.1), respectively. The most prevalent type of dementia was Alzheimer’s disease (48.3%). Twenty-nine participants (48.3%) were diagnosed with depressive disorders, 21 (36.7%) lived alone, 44 (73.3%) were dependent on P-ADL at baseline, and 20 (33.3%) felt lonely often/sometimes. Of the 67 informal caregivers; 38 were partners/spouses, 26 were children, 2 were siblings, and 1 was a niece. All participants except four (two in the intervention group and two in the control group), had informal caregivers participating in the study.

### Follow-up rates in assessments and structured interviews

Follow-up rates of participants throughout the study are presented in Fig 1. Among participants with dementia, the follow-up rates in assessments at 5, 12, 24, and 36 months ranged between 97% and 65%. The corresponding follow-up rates for interviews with caregivers/care staff were 97%–70%. Reasons for missing data during the follow-up period over 36 months were death (n = 17), declined (n = 6), or cognitive impairment (n = 4). In the intervention group, no participant with dementia or caregiver/staff declined assessments. In the control group, there were missing data in six assessments due to decline in the participant with dementia, mainly at 24 and 36 months. Furthermore, in the control group, there were two assessments with missing data at 24 and 36 months because caregivers declined to participate. All participants were able to answer the yes/no questions in the GDS-15 and the PGCMS from baseline to 5-month follow-up. At 12- to 36-months follow-up, there was missing data in four assessments due to an inability to answer the questions due to cognitive impairment. In addition, all participants were able to answer the loneliness questions (multiple-choice answers) up to the 12-month follow-up. At the 24- and 36-month follow-ups there were 46 and 38 assessments of loneliness, respectively, compared with 47 and 39 assessments of GDS and PGCMS, respectively. For the multiple-choice questions regarding social participation posed to the participants with dementia, the number of assessments ranged from 42–43 at 24 months and 33–36 at 36 months.

### Outcomes

Longitudinal changes over 36 months in outcome measures are presented in Tables 2–5.

#### Social participation estimated by caregivers or staff and participants with dementia.

Regarding social participation estimated as occasions per week by caregivers/staff (Table 2), the between group differences (mean, 95% CI) in “taking part in active recreation” and “taking part in organized social activities” was 1.66 (–0.18 to 3.50), *p* = 0.077, and 0.68 (0.00 to 1.37), *p* = 0.050, respectively, in favor of the intervention group at the 12-month follow-up. Despite generally wide confidence intervals, the between-group differences favoring the intervention group remained during the follow-ups, with the exception of the 24-month follow up in “taking part in organized social activities”, where the difference (mean, 95% CI) was −0.47 (–1.18 to 0.24), p = 0.196. In social participation estimated by participants with dementia as often/sometimes during a typical week (Table 4), there was a similar non-significant trend in “taking part in organized social activities“at 12 months with an odds ratio (OR) of 2.254, 95% confidence interval (CI) 0.66–7.69, *p* =0.194, and at 36 months to an odds ratio (OR) of 2.941, (95% CI: 0.63–13.83, *p* =0.172). At baseline, the proportion of the control group estimating “visiting family and friends in their homes” as often/sometimes, was 25/29 (86.2%), whereas in the intervention group, it was 19/31 (61.3%). At the 5-month follow-up, the corresponding proportions were 13/29 (44.8%) and 16/29 (55.2%), which corresponded to an odds ratio (OR) of 4.190 (95% CI: 1.03–17.11, *p* = 0.046) in favor of the intervention group.

According to participants’ satisfaction with the frequency of social participation during a regular week (Table 5), the proportion of those who considered themselves satisfied in “keeping in touch with others through letters, phone, or e-mail” were 87.1% of participants in the intervention group and 89.7% in the control group. At the 5-month follow-up, the corresponding proportions were 69.0% and 93.1%, which corresponded to an odds ratio (OR) of 0.117 (95% CI: 0.01–1.00, *p* = 0.049) in favor of the control group. This trend remained at the 12-month follow-up, where the proportions were 71.4% in the intervention group compared to 92.3% in the control group with an odds ratio (OR) of 0.267 (95% CI: 0.04–1.61, *p* = 0.150). The proportion of participants who were satisfied with experienced frequency in “being visited by family and friends at home” was 64.5% in the intervention group and 82.8% in the control group at baseline. At the 24-month follow-up, the corresponding figures were 59.1% and 90.5%, respectively, with an odds ratio (OR) of 0.161 (95% CI: 0.02–1.39, *p* = 0.097) in favor of the control group. Of those dissatisfied, all wanted to perform the activities regarding social participation more often, except for two questions where they wished to perform two of the activities more seldom at the 5-month follow-up.

#### Loneliness estimated by the participants with dementia.

There were no statistically significant between-group differences in loneliness (Table 5). Twelve participants (38.7%) in the interventions group felt lonely often/sometimes at baseline, compared to eight (27.6%) in the control group. After 36 months, nine participants (45.0%) in the intervention group felt lonely, compared to four (22.2%) in the control group, which corresponds to an odds ratio (OR) of 4.850, 95% CI 0.65–36.49, *p* = 0.125, in favor of the control group (Table 5).

#### Mental health in participants with dementia.

There were no statistically significant between-group differences in the outcomes related to mental health (Table 3). The between-group difference (mean, 95% CI) in depressive symptoms assessed with GDS-15 at five months was –0.82 (–1.98 to 0.35), *p* = 0.169, in favor of the intervention group. The between-group difference (mean, 95% CI) in psychological well-being assessed with PGCMS was –1.00 (–2.40 to 0.41), *p* = 0.163, in favor of the control group at the 24-month follow-up.

## Discussion

This study evaluated the feasibility of a rehabilitation program in terms of follow-up and response rates, and potential short- and long-term effects over three years in participants with dementia, focusing on social participation, loneliness, and mental health. Response rates were high for all assessments of mental health, loneliness, and social participation up to the 12-month follow-up, including questions with multiple-choice options. The response rate decreased after 12 months, particularly for cognitively demanding questions with multiple-choice options. Overall, there were few statistically significant differences between the groups, but some of the findings seem potentially clinically meaningful. Regarding depressive symptoms, there was a difference between the groups after the 20-week rehabilitation period favoring the intervention group, but this difference did not persist throughout the follow-up period. However, there was an indication of increased psychological well-being in favor of the control group after two years. Furthermore, potentially clinically meaningful findings were also seen in active recreation and organized social activities outside of the home in favor of the intervention group, as reported by both participants with dementia and caregivers/staff. The intervention group also reported a higher experienced frequency of visiting family and friends, compared to the control group at five months. Despite this, there were no indications that the rehabilitation program had any clinically meaningful effects on loneliness, mental health (after the 5-month follow-up), or that the intervention group was more satisfied with social participation. At five months, the control group seemed to be more satisfied with their frequency of keeping in touch with others.

Our results highlight that adults with dementia living in the community were able to respond to questions related to mental health and feelings of loneliness over three years in the format of yes/no questions and multiple-choice questions, respectively. Although the mean MMSE for the whole sample had decreased and standard deviation increased over time, indicating progression of the dementia disorder, there were only two and three assessments with missing answers at the 36-month follow-up for yes/no questions and multiple-choice questions, respectively. Participants with missing data in GDS-15 and PGCMS due to cognitive impairment all had MMSE scores below 5. These findings are in accordance with a previous study indicating a challenge to complete yes/no questions related to mental health when the MMSE score is below 5 [43]. However, for those who complete such assessments, earlier studies support that people with severe cognitive impairment can express a meaningful opinion on their quality of life, indicating valid responses [43–45,63].

There were concerns before the study began that participants with dementia might have difficulty answering questions regarding social participation. These questions had multiple-choice answers and posed cognitive challenges such as imagining oneself engaged in activities (e.g., running errands) and subsequently estimating the frequency of such activities, which requires the use of cognitive processes, including memory [36]. The majority of participants were able to respond to these questions for up to 12 months. However, there was an increase in missing data after this period, which may be attributed to disease progression. The preplanned strategy to gather information about the frequency of participation in each question from the caregivers was valuable in avoiding data loss. Furthermore, no participants in the intervention group declined to participate in the four follow-ups. In contrast, a few participants in the control group declined follow-up, particularly at the 24- and 36-month follow-ups. This might have been minimized had we offered an attention control activity, which might have increased motivation to participate in follow-up assessments.

Since this is a randomized controlled pilot study that lacks statistical power, we cannot draw conclusions about the effects of the rehabilitation program. However, we did observe clinically meaningful findings. There were overall non-significant clinically relevant differences between the groups, where the intervention group performed active recreation a mean of 1.66 times more than the control group per week and organized social activities a mean of 0.68 times more/week at twelve months. These results persisted over time in favor of the intervention group according to assessments made by caregivers/staff and largely corresponded with the assessments made by the participants. These differences may have clinical significance. For example, the Finnish Geriatric Intervention Study to Prevent Cognitive Impairment and Disability (FINGER) has shown that social and physical activities may be important interventions for promoting health, including improving or maintaining cognitive function in older adults at risk of developing dementia [64,65]. In terms of physical activity, specifically, studies involving individuals with Alzheimer’s disease have reported that social and physical activities can enhance one’s sense of self, reduce anxiety, and promote overall well-being [66]. Participation in organized physical and social activities has been shown to foster a sense of togetherness, increased activity, gratitude, and overall well-being among people with dementia [38,67]. The potentially positive findings in these areas may be due to this being one of the focus areas in the rehabilitation program. During the 20-week intervention period, efforts were made to encourage participants to attend the sessions at the day-rehabilitation unit, as well to introduce them to activities outside of home according to their individual goals. These efforts may have strengthened participants with dementia in overcoming barriers for leaving the home, and fostered motivation to participate in activities in society. In the qualitative study by Lindelöf et al. (2023), staff expressed that providing support led to increased self-esteem among participants, who then began expressing their own wishes and trying new activities [37]. In the study by Sondell et al. (2021), it was found that the participants with dementia themselves wished to remain active after the 20-week rehabilitation period [38]. Additionally, as reported by Hasselgren et al. (2024), measurements of physical activity levels in the MIDRED study indicated that the intervention group was more physically active in their everyday lives and spent less time being sedentary compared to the control group over a 36-month span [39]. Together, these studies highlight significant findings that warrant further investigation through a full RCT to accurately assess the effects.

Although non-significant, there was an indication of a possible clinically relevant difference between the groups in depressive symptoms at the 5-month follow-up in favor of the intervention group. As depression constitutes a major problem in the group, and pharmacological treatments may have no effect [29], future research in this area is needed. The indication of a non‑significant increase in psychological well‑being in the control group after two years may be linked to reported increases in satisfaction and in the caregiver/staff‑rated frequency of ‘being visited by family and friends at home’ at 24 months. However, this indication should be interpreted with caution due to the loss of participants later in the study. The rehabilitation program seemed to have no impact on participants’ satisfaction with social participation or feelings of loneliness in the intervention group. This was despite indications of increased frequency of social participation. Previous literature has shown that external social activities in the community are associated with less loneliness among older adults [68]; however, this result may not be transferable to older adults with dementia. The intervention to support social participation was mainly focused on support in participation in exercise groups or gyms, and in social activities, in the community [39]. There may be other aspects of social participation that are more important affecting loneliness in this group. In Lampinen et al. (2021), an association was seen between loneliness and the desire for more friends, as well as meeting family and friends more often among very old people with and without dementia [51]. This might indicate a need for increased social interaction with close relations, such as family and friends, to impact loneliness, and could be an important focus of rehabilitation*.* Further, the MIDRED study was a research project, and thus team staff were not available for the participants between the 20-week intervention and the follow-up periods. As a result, participants may have experienced feelings of abandonment and loneliness, especially given their situation of living with a progressive illness such as dementia. This situation coincided with the outcome assessments conducted at 5 months. The results indicate the control group as more satisfied with frequency of keeping in touch with others through letters, phone or e-mail at 5 months. An interpretation based on the proportions at 5 months may be that the intervention group had become less satisfied, possibly explained by the fact that they had just finished an intensive 20-week period of rehabilitation with activities outside of home, as well as frequent contacts with team staff and participants in the study. It may be valuable to offer continuous contact with the team but at a lower intensity between the intervention periods (20-week period and the two follow-ups). In addition, WHO (2021) recommends regular follow-up appointments with a fixed contact in healthcare when living with a lifelong disease like dementia [69]. Performing an assessment of loneliness during the ongoing intervention would also be interesting to gain a deeper understanding of this area.

A strength of this study is the follow-up over three years, which enables us to evaluate the questions and assessments, as well as follow the health status of the participants with dementia over time. A limitation is the increasing amount of missing data over three years, mainly due to mortality, which may introduce bias when evaluating potential effects. Considering the mortality rate when calculating sample sizes in future randomized trials with long follow-up periods in this population is important. The response rates for GDS-15 and PGCMS were high, and the literature supports the use of these scales among older people with cognitive impairment [43]. However, there may be a limitation in these scales in detecting differences over time. The sample in the study was heterogenous concerning cognitive function and dementia type. This reflects the population studied; however, it limits possibilities to draw conclusions regarding feasibility based on these aspects. All participants were recruited from a limited area in northern Sweden which limits the external validity. Fortunately, the three-year follow-up was completed during the first months of 2019, and thus before the COVID-19 pandemic.

## Conclusion

The assessments used in this study over three years, in the areas of social participation, loneliness and mental health seem feasible for further use. It seemed cognitively demanding for participants with dementia to answer questions regarding social participation over a three-year span. Therefore, the strategy to also ask informal caregivers/staff, especially after 12 months, was valuable to avoid data loss. The results also indicate that person-centered multidimensional interdisciplinary rehabilitation for community-dwelling older adults with dementia can produce potentially clinically meaningful findings on social participation and mental health (short-term). Based on the results from this study as well as the earlier published studies from the MIDRED study [37–40], it seems relevant to proceed further to an adequately powered RCT and conducted in additional geographical regions. To alleviate loneliness among older adults with dementia, the intervention may need to be developed to also support social interaction with close relations, such as family and friends, as well as to assess loneliness during ongoing intervention to explore the potential effects.

## Supporting information

S1 File CONSORT-checklist J Lampinen. (DOCX)

S2 File Study protocol J Lampinen translated to English. (PDF)

S3 File Study protocol J Lampinen original language swe. (PDF)
