# Supplementary material for: An interdisciplinary rehabilitation program for adults with dementia—A randomized controlled pilot trial evaluating social participation, loneliness and mental health
Source: PLoS One. 2026 Mar 24;21(3):e0345518. doi: 10.1371/journal.pone.0345518 (PMC13012523; doi:10.1371/journal.pone.0345518)
Supplement: S2 File — (PDF) [file pone.0345518.s002.pdf]

Application for Ethical Review

Decision Date: 2013-03-04

Application for Ethical Review of Research Involving Humans

Information for the application, see Guidance for the application (www.epn.se)

Depending on the type of research the application concerns, the requested information will have varying relevance. For changes to previously approved applications, see Guidance for the application.

To the Regional Ethical Review Board in: Umeå
The regional ethical review board to which the research principal belongs, see respective board (www.epn.se).

Fee paid date: 2015-08-14
Please note that an application is never complete and can therefore not be processed until the form is correctly filled out and the fee is paid.

Project Title:
Person-centered multidimensional interdisciplinary rehabilitation of elderly people with dementia living in ordinary housing, including education and support for relatives: a randomized controlled study

Provide a descriptive title in Swedish for laypersons. The title should not contain confidential information. Also specify, if applicable, e.g., in clinical drug trials, the project's identity, research plan/protocol number, version, date. For changes to previously approved applications, see Guidance for the application.

Project Number/Identity:
Version Number:
EudraCT Number (for drug trials):

Information to be filled in by the regional ethical review board
Application complete:
Dnr
Request for additional information (in substance):
Requested information received:
Decision date:
Expedition date:

The application concerns (also applies to requests for advisory opinions):
Research involving only one research principal (5,000 SEK) √
Research involving more than one principal (16,000 SEK)
Research involving more than one research principal, but where all research subjects or research

objects have an immediate connection with only one of the research principals (5,000 SEK)
Only processing of personal data (5,000 SEK) (When only existing personal registers are used, e.g., national databases)
Research concerning clinical drug trials (16,000 SEK)

Change of previously approved application according to §4 of the ordinance (2003:615) on ethical review of research involving humans (2,000 SEK)

If the board finds that the research project does not fall within the scope of the ethical review law, an advisory opinion is requested. (See §§4a and 4b of the ordinance 2003:615 and Guidance for the application)

Yes: **√**
No:

1. Information about the research principal

1:1 Research Principal (See p. 1:1 in Guidance for the application)

The application for ethical review of research must be made by the research principal. The research principal refers to a government agency or a physical or legal person in whose activities the research is conducted.

Name: Umeå University

Address: 901 87 Umeå

1:2 Authorized Representative of the Research Principal

The authorized representative is e.g., head of department, unit manager, operations manager. The research principals themselves determine, through internal work and delegation orders or through power of attorney, who is authorized to represent the research principal.

Name: Ann Sörlin

Title: Head of Department

Address: Umeå University, Vårdvetarhuset, 901 87 Umeå

1:3 Researcher responsible for the implementation of the project (contact person) (See p. 9 bil. nr 10 and p. 1:3 in Guidance for the application)

Note! The principal investigator is responsible for ensuring that other participants who will carry out the project have sufficient competence (scientific and clinical) and, in drug trials, have sufficient knowledge of "Good Clinical Practice" (GCP). In doctoral studies, the supervisor is usually the principal investigator.

Research protocol as approved by ethical committee- translated from Swedish.

Name: Håkan Littbrand

Title: FoAss

Address: Umeå University, Department of Geriatrics, 901 87 Umeå

Email: hakan.littbrand@umu.se

Phone: 090-785 87 12

Mobile: 0730-444 851

1:4 Other Participants (See p. 9 bil. nr 1 and p. 1:4 in Guidance for the application)

Other participating research principals and researchers responsible for locally implementing the project (contact persons) are listed here or in an appendix with names and addresses (see p. 9 bil. nr 1).

Yngve Gustafson, Professor and Chief Physician. Head of the Department of Community Medicine and Rehabilitation, Department of Geriatrics, Umeå University.

Mia Conradsson, Postdoctoral Fellow and Licensed Physiotherapist. Department of Community Medicine and Rehabilitation, Department of Geriatrics, Umeå University.

Peter Nordström, Professor and Chief Physician. Department of Community Medicine and Rehabilitation, Department of Geriatrics, Umeå University.

Maine Carlsson, Doctor of Medicine and Dietitian. Geriatric Center, Norrlands University Hospital

Birgitta Olofsson, Associate Professor and Licensed Nurse. Department of Nursing, Umeå University.

Nina Lindelöf, Postdoctoral Fellow and Licensed Physiotherapist. Department of Community Medicine and Rehabilitation, Department of Physiotherapy, Umeå University.

Ingeborg Nilsson, Associate Professor and Licensed Occupational Therapist. Department of Community Medicine and Rehabilitation, Department of Occupational Therapy, Umeå University.

1:5 Report access to necessary resources during the project's implementation (See p. 9 bil. nr 9 and p. 1:5 in Guidance for the application)

Specify who is responsible (head of department, operations manager, or equivalent) for the safety of research subjects at all units/clinics where research subjects will participate. Certificates from these responsible persons must be attached (see p. 9 bil. nr 9). The certificate should indicate that the necessary financial, structural, and personnel resources are available to ensure the safety of research subjects.

Ann Sörlin, Head of the Department of Community Medicine and Rehabilitation, Umeå University (Appendix 9a).

Undis Englund, Operations Manager at the Geriatric Center, Norrlands University Hospital (Appendix 9b).

1:6 Application/notification to other authorities in certain cases (see p. 1:6 in Guidance for the application)
Sent Date
a) In clinical drug trials: Medical Products Agency
b) In the establishment of a biobank: National Board of Health and Welfare
c) In studies involving ionizing radiation: Radiation Protection Committee

2. Information about the project

2:1 Summary description of the research project (See p. 9 bil. nr 2 and p. 2:1 in Guidance for the application)

The description should be understandable to all members of the board. Therefore, avoid terminology that requires specialized knowledge. Specify the background and purpose of the study and the scientific question(s) being addressed. Specify the main research variables. Describe the expected knowledge gains from the project and their significance. Specify whether it is a registry study, commissioned research, etc. Detailed information for professionals about the research plan/protocol (program) should be attached as an appendix (see p. 9 bil. nr 2). For the design of the research plan/protocol, see p. 2:1 in Guidance for the application. Specify when data collection is expected to be completed. A more detailed description of the study's implementation intended for laypersons can be attached to the mandatory research plan for professionals if needed.

Background:

In Sweden, there are currently about 160,000 people with dementia, and including relatives and friends, a total of about one million people are affected. Dementia can cause significant suffering as it is a disease that affects the entire life situation of those affected and their relatives. Dementia has a progressive course and is the leading cause of dependence in activities of daily living (ADL) among elderly people. Within 2-3 years, about half of those diagnosed have moved to a special housing.

Dementia causes a gradual decline in cognitive function, which includes memory, attention, learning, and problem-solving. In addition, the consequences of dementia can be many and complex. Impaired balance and walking ability are common, which, together with impaired cognition, increases the risk of falls and fall injuries. Impaired mobility, along with difficulties in orientation and reduced initiative, means that the person with dementia is also at risk of having few social contacts and low physical activity levels. Studies have shown that ADL ability, cognitive function, and well-being among people with dementia can be influenced by interventions with physical or social activity. This indicates the possibility of influencing the course and the importance of maintaining these functions for as long as possible. Other aspects of the complex problem are that people with dementia may have reduced physiological reserve capacity, which means an

increased risk of developing delirium from internal and external disturbances, such as pain or environmental factors, and a greater risk of complications from illness or medication-related problems. Furthermore, people with dementia also have an increased risk of other medical conditions such as malnutrition, incontinence, poor oral health, and depression. In addition to depression, symptoms such as anxiety, aggression, restlessness, hallucinations, wandering behavior, and sleep disturbances are common. These symptoms are often referred to as behavioral and psychological symptoms of dementia, BPSD. BPSD causes significant suffering for the person with dementia, but also for relatives, and is the most common reason for the person with dementia moving to special housing. BPSD, cognitive impairment, and the need for physical help in daily life affect the extent of the efforts from a caring relative and can negatively impact the relative's mental and physical health, quality of life, economy, and participation in society.

Many of the complications associated with dementia are preventable or modifiable. The complex nature of dementia highlights the need for person-centred, multidimensional, interdisciplinary rehabilitation to support individuals in regaining or maintaining optimal functional ability, as well as enabling independent living and active participation in society. Person-centred, multidimensional, interdisciplinary rehabilitation refers to a process in which a team of professionals from various disciplines conducts a comprehensive assessment to identify the individual's problems and needs, as well as strengths and resources. The team, together with the person with dementia and their informal caregiver(s), jointly agrees on interventions and goals for the rehabilitation period. Progress is regularly evaluated through scheduled meetings. This model is currently used in clinical practice within geriatric rehabilitation. Systematic reviews by the Swedish Agency for Health Technology Assessment and Assessment of Social Services (SBU) and meta-analyses from The Cochrane Collaboration have concluded that this approach is effective in the care and rehabilitation of older adults with complex needs in inpatient settings. Reported benefits include improvements in activities of daily living (ADL) and a higher likelihood of remaining in ordinary housing after hospital discharge. However, the model has not yet been specifically evaluated for people with dementia in outpatient settings.

An important complement to rehabilitation for people with dementia is to provide education, support, and counselling for informal caregivers. Studies have shown that such interventions may reduce caregiver burden, depressive symptoms, and negative health impacts, and may also extend the time the person with dementia is able to remain living in ordinary housing.

Aim and Implementation

This research project aims to evaluate the effects of a person-centred, multidimensional, interdisciplinary rehabilitation programme for community-dwelling individuals with dementia. The programme includes educational and supportive interventions for their informal caregivers.

Participants will be randomly assigned to receive either usual care or a 16-week person-centred rehabilitation programme, including two follow-up assessments at five and fifteen months post-intervention. The **primary research question** is whether the intervention can mitigate the consequences of dementia and thereby increase the proportion of individuals who continue living in ordinary housing two and three years after the intervention begins.

Secondary research questions include whether the programme:

- reduces perceived caregiver burden, improves caregiver health-related quality of life, and prevents depressive symptoms;

- increases social participation and reduces depressive symptoms and diminished well-being in people with dementia;
- prevents or slows negative outcomes associated with dementia, such as physical inactivity, cognitive and physical decline, increased need for assistance with ADL, behavioural and psychological symptoms of dementia (BPSD), falls and fall-related injuries, malnutrition, and inappropriate medication use;
- is cost-effective;
- is feasible, based on participants' and caregivers' experiences, attendance rates, and any adverse effects;
- differs in implementation and outcomes based on sex, age, presence of a caregiver, cognitive status, and the presence of BPSD.

Data collection is expected to be completed by November 2020 (see Research Plan, Appendix 2).

Significance

The overarching goal of the project is to improve care for people with dementia. Unlike other neurodegenerative conditions such as Multiple Sclerosis (MS) or Parkinson's disease, it is currently uncommon for Swedish healthcare providers to offer structured rehabilitation periods specifically for dementia.

Dementia is one of the most resource-intensive chronic conditions, with an estimated annual cost of approximately SEK 63 billion in Sweden. Given the expected dramatic increase in dementia prevalence in the coming decades, the WHO recommends that dementia be prioritised as a global public health concern.

The complexity of dementia care indicates a need for person-centred, multidimensional, interdisciplinary rehabilitation to achieve optimal outcomes — namely, to restore or maintain the highest possible level of functioning and promote autonomy and participation in society. The current scientific evidence supporting this type of rehabilitation for individuals with dementia is limited.

This intervention is expected to have positive effects on dementia-related outcomes, including decline in ADL and physical functioning, BPSD, social isolation, depression, and caregiver burden — all of which may influence the ability to remain in ordinary housing. Positive findings could have substantial clinical implications, providing a model of care to reduce suffering caused by dementia. Moreover, delaying the need for institutional care could lead to significant societal cost savings.

See also the attached research plan (Appendix 2).

2.2 Which scientific question(s) form the basis for the design of the project?
If the project can be characterised as hypothesis-testing, please state the primary and, if applicable, secondary hypotheses.
Reference to more detailed information for professionals may be made in the attached research plan/protocol according to section 2.1.

Primary Hypothesis:
The primary hypothesis is that a person-centred, multidimensional, interdisciplinary rehabilitation programme for individuals with dementia living in ordinary housing, which includes education, support, and counselling for informal caregivers, increases the proportion of individuals who are alive and remain in ordinary housing after two years (primary outcome variable).

Secondary Hypotheses:
The programme is hypothesised to:

- Reduce the perceived burden among informal caregivers of individuals with dementia, while improving their health-related quality of life and mitigating symptoms of depression.
- Enhance social participation and counteract depression and reduced well-being among individuals with dementia.
- Prevent or slow the progression of dementia-related consequences such as physical inactivity, decline in cognitive and physical functioning, increased need for assistance with activities of daily living, occurrence of behavioural and psychological symptoms of dementia, falls and fall-related injuries, malnutrition, and reduce the use of inappropriate medications among individuals with dementia.
- Be cost-effective.
- Be feasible for individuals with dementia and their caregivers, as indicated by a positive experience of participation, high attendance rates, and the absence of serious adverse events or discomfort related to the intervention.

2.3. Describe the results of relevant animal studies (applies to clinical treatment research). If no animal studies have been conducted, please provide the reasons for this.

Not applicable

2. 4. Provide an overview of the study procedures, data collection, and the nature of the data.

The description should clearly outline how the project is planned to be carried out. Describe the nature of the data to be collected and how the reliability of the data will be ensured (e.g. quality control/monitoring). For questionnaires and interviews, the methodology should be described, including the content of questions and how conclusions will be drawn. Questionnaires and rating scales must be attached (see Section 9, Appendix 5). For medical research, include information such as types of interventions, measurement methods, number of visits, time required for each session, dosages and routes of administration of any drugs and/or isotopes, and the amount of blood samples taken (including total volume in case of repeated sampling). State whether and how the study procedures differ from standard clinical practice. If a treatment is being studied in humans for the first time, this must be clearly stated and relevant safety procedures described. Also, describe the procedure for providing any necessary treatment after the study has ended. Describe the procedure for the collection of biological material. Include information on data sources and procedures for the processing of personal data. More detailed information may be provided in the attached research plan.

Intervention

Participants will be randomly assigned to one of two groups—control or intervention—after completion of baseline assessments. Allocation to the control group means that participants will receive standard care and support. Allocation to the intervention group means that participants with dementia will be enrolled as patients at the outpatient unit of the Geriatric Centre, University Hospital of Northern Sweden. The intervention will be delivered at the Geriatric Centre in facilities suitable for day rehabilitation activities, although home-based or community-based interventions may also be provided depending on individual rehabilitation goals.

An interdisciplinary team—comprising a physician, nurse, assistant nurse, physiotherapist, occupational therapist, social worker, dietitian, neuropsychologist, dental hygienist, and pharmacist,

all experienced in the rehabilitation of older adults with cognitive and physical impairments—will collaborate in the assessment and treatment of each participant.

Each participant will be assessed across the following potential problem areas associated with dementia: functional physical capacity, cognitive function, activities of daily living (ADL), fall risk, social participation, physical activity, nutrition, comorbidities, behavioural and psychological symptoms of dementia (BPSD), and medication use. Based on identified problems, as well as strengths, resources, and personal preferences, an individualised team will be formed around each participant. The team will agree on specific interventions and individual rehabilitation goals. The social worker will assess the need for individual support and counselling for informal caregivers.

After the 16-week intervention period, a handover will be conducted to the primary care centre, which will resume medical responsibility for the participant with dementia.

Intervention Components for Persons with Dementia (16 weeks)

1. **Physical Activity**: Individually tailored functional training sessions (45 minutes, twice weekly) with a physiotherapist, aiming to improve muscle strength, balance, and mobility. The training is based on the High-Intensity Functional Exercise (HIFE) Program. Individualised advice will be provided to ensure at least 150 minutes of moderate-intensity physical activity per week, in line with current health promotion guidelines.
2. **Individual Goal-Oriented Interventions**: Up to two sessions per week based on the participant's individual rehabilitation goals, delivered by the appropriate professional within the team. Interventions are grounded in evidence-based practice and clinical experience.

Intervention Components for Informal Caregivers (16 weeks)

1. **Group Sessions**: Six group meetings will be offered, including information and discussions on specific themes tailored to the needs and preferences of the participants. Topics may include the progression of dementia, management and prevention of BPSD, health-promoting activities, and available community services. The social worker will assess caregivers' needs and preferences prior to the intervention and will co-facilitate the sessions, also encouraging social interaction among participants.
2. **Support and Counselling as Needed**: Caregivers will be offered up to six individual sessions with a social worker during the intervention period. Support may include guidance on formal care options, financial assistance, psychological support, and strategies for managing BPSD.

Follow-Up

The intervention will be followed up at two time points: five and fifteen months after the end of the rehabilitation period. The interdisciplinary team will assess the extent to which the person with dementia has met the health promotion recommendations regarding physical activity and achieved their individual rehabilitation goals. Interventions will be adjusted as needed. Follow-up for caregivers will be conducted by the social worker, who will provide further guidance and support if necessary.

Data Collection

Participants in both the intervention and control groups will undergo assessments at baseline (study inclusion), and at 4 (end of rehabilitation), 12, 24, and 36 months. Assessments will include rating scales and interview-based questions, conducted during a home visit by an assessor blinded to group

allocation. The visit for the person with dementia is expected to take approximately two hours and may be split into two sessions if needed. The caregiver visit will take about one hour. Additional data on the person with dementia will be collected from medical records. The assessment tools and rating scales used are reliable and valid, and those used for persons with dementia are appropriate for individuals with cognitive impairment. All assessments will be conducted by trained personnel blinded to group allocation.

Primary outcomes

Proportion of people with dementia who still is living at home, which is the inverse of death or institutionalisation combined (describes the odds of someone being alive and in their home at a point in time), at the 24- and 36-month follow-up assessment.

Secondary outcomes

Primary caregivers:
Caregiver burden using Caregiver Burden Scale.
Depressive symptoms using Geriatric Depression Scale (GDS-15).
Health related quality of life assessed using the SF-36 (Health survey).

Persons with dementia:
Depressive symptoms using Geriatric Depression Scale (GDS-15).
Psychological well-being using Philadelphia Geriatric Center Morale Scale (PGCMS).
Participation in the society by questions concerning number of visits in the home, number of visits made to others and contacts with relatives and friends using telephone or other media.
Physical activity using IPAQ-E (International Physical Activity Questionnaire–Elderly) including questions added by the authors.
Cognitive function using Mini-Mental State Examination (MMSE), Alzheimer's Disease Assessment Scale-Cognitive Subscale (ADAS-cog), and Verbal fluency.
Functional capacity using the Berg Balance Scale, chair-stand test, and gait speed test over 2.4 meters.
ADL performance using Functional Independence Measure (FIM), Lawton scales (P- and IADL).
BPSD using Neuropsychiatric Inventory (NPI).
Nutritional status using Mini Nutritional Assessment (MNA).
Inappropriate drugs including interactions using The National Board of Health and Welfare recommendations,
http://www.socialstyrelsen.se/Lists/Artikelkatalog/Attachments/18085/2010-6-29.pdf
Incidence of falls and fall-related injuries collected from medical records and records in the municipality.
Feasibility of the intervention by semi-structured interviews of people with dementia and primary caregivers concerning the experiences of participating in the intervention, analysed according to Granheim & Lundman (64), and by registering the attendance and adverse events.
- The research team will document any adverse events or discomfort experienced during the intervention in a standardized protocol. Relatives and staff will be regularly asked whether the participant has shown any signs of discomfort related to traveling to or staying at the Geriatric Center. At each training session, the physiotherapist will record any negative or positive events and reactions,

the participant's level of motivation, and the achieved training intensity using a protocol previously employed in exercise studies involving individuals with dementia conducted by the research group.

The participants' experiences of the intervention will also be explored through interviews with 15 individuals with dementia and their respective relatives. The opening question for relatives will be: *"How do you experience the support you have received from the Geriatric Center?"* A follow-up question will be asked to all participants: *"Is there anything you would like to change?"* Interviews with individuals with dementia will be conducted in connection with a visit or a training/activity session at home to facilitate recall and contextual relevance. If necessary, the interviews may be conducted in two sessions. The opening question will be: *"How do you experience the activities you have participated in?"* A follow-up question will be asked to all: *"Is there anything you would like to change?"* The interviews will be analyzed using qualitative content analysis.

Cost-effectiveness will be analyzed using the EQ-5D instrument alongside calculations of the costs associated with the intervention, healthcare utilization, and social services. Healthcare and social service consumption will be measured by recording the number of inpatient hospital days and outpatient visits through a review of medical records. Additionally, both formal and informal care will be documented using the Resource Utilization in Dementia (RUD) instrument.

Comorbidities and mortality will be monitored throughout the study period (36 months) via medical record review.

The data sources used in the study will include the participants' medical records from municipal services and Västerbotten County Council, as well as the Apo-dos registry.

All data will be handled confidentially. Upon inclusion, each participant will be assigned a unique code number. In the data files used for analysis, all material will be de-identified, and only the participant's code number will be recorded—no personal identification numbers will be included. Code lists will be stored separately from the data files in fireproof storage accessible only to authorized researchers.

Documentation of the intervention activities will be recorded on paper-based protocols provided by the project.

2.5 Describe whether collected biological material will be stored in a biobank.

A biobank refers to biological material from one or more individuals that is collected and preserved either indefinitely or for a defined period, and where the origin of the material can be traced back to the individual(s) from whom it was obtained. Specify where and how the samples to be stored will be kept, coding procedures, and the conditions under which samples may be released. Also indicate the responsible entity (custodian) for the biobank.

Not applicable.

2.6 Documentation, data protection, and archiving

Describe how study procedures and any interventions will be documented. Specify whether audio or video recordings will be used. If material is to be coded, describe the coding procedure, who stores the code lists/keys, who will have access to them, and where and for how long they will be stored. Also state whether the material will be anonymised or destroyed.

Describe the level of accessibility to the data and how it will be stored, as well as how necessary confidentiality and data protection will be ensured.

Data Management and Documentation

Research protocol as approved by ethical committee- translated from Swedish.

Information collected during testing sessions will be documented using paper-based protocols, which will be stored securely with access restricted to project personnel only. Upon inclusion in the study, each participant will be assigned a unique identification code. After data collection, the data will be entered into statistical software, and audio recordings from interviews will be transcribed into text files. No video recordings will be used in the study.

All data files used for analysis will be de-identified, and only the participant's code number will be recorded—personal identification numbers will not be included in the data files. During transcription, participants will also be de-identified. Qualitative content analysis, a well-established research method, will be used to analyse the interview data (qualitative data).

Codebooks, paper protocols, and audio files will be stored separately from the entered data files and archived in a fireproof, locked facility accessible only to authorised researchers. Electronic data files and transcribed text files will be backed up on a server provided by Umeå University. The creation date of each electronic file will be recorded.

All research materials will be retained for a minimum of ten years following the final scientific publication from the project.

2.7 Describe previous experience (your own and/or others') with the procedure, technique, or treatment being used.

It is particularly important to clearly present any known risks or complications, and where applicable, to reference relevant publications. For new treatments involving patients (e.g. pharmacological treatments), indicate how many patients (with the current or other conditions) have previously received the proposed treatment, the dosage of the drug (or other dosage), and the duration of treatment that has been studied.

Research Team and Expertise

The study will be conducted by researchers at Umeå University, Department of Community Medicine and Rehabilitation (Units of Geriatrics, Physiotherapy, and Occupational Therapy), and the Department of Nursing. These units have extensive experience in conducting and analysing results from intervention studies involving frail older adults with cognitive and physical impairments (Jensen et al., *Ann Intern Med* 2002;136:733–741; Rosendahl et al., *Aust J Physiother* 2006;52:105–113; Littbrand et al., *J Am Geriatr Soc* 2009;57:1741–1749; Lundström et al., *J Am Geriatr Soc* 2005;53:622–628).

The research group also has substantial experience in both clinical practice and research involving interdisciplinary teamwork (Lundström et al., *J Am Geriatr Soc* 2005;53:622–628; Stenvall et al., *Arch Gerontol Geriatr* 2012;54:e284–289; Jensen et al., *Ann Intern Med* 2002;136:733–741). The exercise programme to be used in the study—the High-Intensity Functional Exercise (HIFE) Program—was developed by the research group specifically to be feasible for individuals with cognitive and physical impairments. The HIFE Program has been evaluated in residential care settings, including among individuals with dementia (Littbrand et al., *Phys Ther* 2006;86:489–498).

No serious adverse events resulting in lasting harm or illness have been associated with the exercise programme. Participants with dementia have been able to complete the training in a manner comparable to those without dementia, in terms of attendance, exercise intensity, and reported discomfort.

3. Information about research participants

3.1 How are research participants selected?

A research participant refers to a living person who is the subject of the research. Describe the selection criteria (inclusion and exclusion). Explain how the researcher will come into contact with or identify suitable participants. If recruitment is done via advertisements, the advertisement material must be submitted as an appendix (see Section 9, Appendix 3). If the study involves children or individuals who are temporarily or permanently unable to provide informed consent, this must be specifically justified. Similarly, if certain groups are excluded from participation, this must be specifically explained.

Study Setting and Recruitment

The study will be conducted at the Geriatric Centre, University Hospital of Northern Sweden in Umeå. Participants will be recruited from Umeå Municipality and surrounding municipalities.

Inclusion criteria for participants with dementia include: a confirmed diagnosis of dementia, age 60 years or older, living in ordinary housing, a score of 10 or higher on the Mini-Mental Test (MMT), approval from the responsible physician to participate in the study, no initiated transition to residential care (i.e., not on the municipal waiting list for residential care or receiving respite care), an expected survival of more than six months, the ability to stand up independently or with the support of armrests and/or one person, and sufficient hearing and Swedish language skills to enable participation in assessments.

Inclusion criteria for informal caregivers include: being a person responsible for the care and support of the individual with dementia. A maximum of two caregivers per participant with dementia will be invited to participate. The term "informal caregiver" includes family members, relatives, and others such as neighbours or friends who provide support.

Participants will be recruited through primary care centres and the outpatient clinic at the Geriatric Centre, University Hospital of Northern Sweden. Based on project guidelines, potential participants will be identified by nurses or physicians at the primary care centres (preferably those responsible for dementia care) or at the Geriatric Centre. These healthcare professionals will make the initial contact and ask whether the potential participant consents to being contacted by the research team. Written information will be sent to the potential participant, followed by verbal information. Information and invitations to caregivers will be provided in conjunction with the inclusion of the person with dementia. Additional recruitment will be conducted through local branches of the Swedish Alzheimer's Association in Umeå and surrounding municipalities. Interested individuals will be asked to contact the research team (see information letter, Appendix 4b). No public advertising will be used for recruitment.

Clinical experience and previous research support the notion that individuals with dementia who score at least 10 on the Mini-Mental Test are capable of expressing interest in participating in an activity and can reliably report their experiences of testing or training (Mowley et al., *Int J Geriatr Psychiatry* 1999;14:776–783; Hoe et al., *Age Ageing* 2005;34:130–135; Littbrand et al., *Phys Ther* 2006;86:489–498; Conradsson et al., *Aging Ment Health* 2013;17:638–645). However, individuals with dementia may not always fully comprehend the overall purpose and scope of the intervention or study. In cases where the participant expresses willingness to participate, consultation with the next of kin, legal guardian, or trustee (if applicable) will be required to ensure informed decision-making.

The study is expected to provide direct benefit to the participants and contribute to findings that may benefit others with the same or similar conditions. The research is anticipated to involve minimal risk of harm and minimal discomfort for the participants.

Research protocol as approved by ethical committee- translated from Swedish.

3.2 Describe the relationship between the researcher/investigator and the research participants

☒ Therapist (e.g., physician, psychologist, physiotherapist) – research participant (e.g., patient, client)

Instructor (teacher) – student

Employer – employee

Other relationships that may potentially involve a risk of undue influence. Please describe:

3.3 Describe the statistical basis for determining the size of the study population(s)/study material.
Present the statistical power calculation, or provide equivalent considerations that clarify the study's ability to adequately answer the research questions.

Within 2–3 years of receiving a dementia diagnosis, approximately half of the individuals have transitioned to residential care (SBU – The Swedish Council on Technology Assessment in Health Care. *Dementia – Etiology and Epidemiology*, 2008).

A power analysis (80% power, two-sided test, α = 0.05) was conducted based on an expected difference between the intervention and control groups at the 24-month follow-up. The anticipated difference is 50% in the proportion of participants who are alive and living in ordinary housing (75% in the intervention group versus 50% in the control group), which corresponds to the inverse of the combined outcome of death or transition to residential care.

The analysis indicates a required sample size of 179 participants with dementia, including an estimated 5% attrition rate over the course of the study.

3.4 Could the research participants be enrolled in multiple studies simultaneously or in close proximity to this one? If so, what type of research?
(See Section 3.4 in the Application Guide)

The research participants will not be enrolled in any additional studies, to the best of our current knowledge.

3.5 What insurance coverage is in place for the research participants involved in the project?
It is the responsibility of the research principal (sponsor) to ensure that insurance is in place to cover any potential harm or injury that may occur in connection with the research.

Participants with dementia are covered by patient insurance upon enrolment at the Geriatric Centre, University Hospital of Northern Sweden. No specific insurance has been arranged for informal caregivers included in the study, as they do not receive any form of medical treatment.

3.6 What financial compensation or other benefits are provided to the research participants involved in the project, and when is the compensation paid?
A more detailed description may be provided in an appendix

Compensation for discomfort and inconvenience. Indicate amount (before tax):
Compensation for lost income: Yes ☐ No ☑
Travel reimbursement: Yes ☑ No ☐
Waiver of medication costs: Yes ☑ No ☐
Waiver of other costs. Please specify:
Research participants with dementia will have their patient fees covered.
Other benefits. Please specify:

When is the compensation paid?
Patient fees and healthcare travel costs are invoiced directly to the project. Travel reimbursement for participating caregivers is paid upon submission of supporting documentation.
No direct financial compensation is paid to participants.

4. Information and consent

4.1 Procedure and content of the information provided when research participants are asked to participate

According to Section 16 of the Swedish Act concerning the Ethical Review of Research Involving Humans (2003:460), research participants must be informed about the overall plan of the research, the purpose of the study, the methods to be used, potential consequences and risks, the identity of the research principal, that participation is voluntary, and that they have the right to withdraw at any time without providing a reason. Describe how and when this information is provided and what it includes. Specify who provides the information. Normally, a brief and easily comprehensible written information sheet should be provided. This written information must be attached to the application (see Section 9, Appendix No. 4). If no or only partial information is given, the reasons for this must be clearly stated.

Potential participants—both the individual with dementia and their informal caregiver—will receive written information (see Appendices 4a and 4b for the person with dementia, and Appendix 4c for the caregiver), as well as verbal information containing the same content, with the opportunity to ask questions. The written information will be sent to the participant prior to a visit from a project representative, who will then provide the verbal information in person (see also recruitment description under section 3:1).

4.2 How and from whom is consent obtained?

Describe the procedure: who obtains consent, when this occurs, and how the consent is documented. A detailed description is particularly important when the study involves children or individuals with impaired decision-making capacity, as well as when studying groups such as school classes, associations, organizations, companies, religious communities or congregations, or groups that interact through social media.

Verbal informed consent to participate in the project will be obtained from the participant and documented by project staff, after both written and verbal information has been provided and any questions have been answered (see Appendix 4d). Participants may withdraw from the study at any time without providing a reason. They may also choose to decline participation in specific parts of the study or intervention, such as individual assessments or activity sessions.

Clinical experience and previous research support that individuals with dementia who have a cognitive function corresponding to at least 10 points on the Mini-Mental Test can express interest in participating in an activity and reliably report their experience of testing or training (Mowley et al., *Int J Geriatr Psychiatry* 1999;14:776–783; Hoe et al., *Age Ageing* 2005;34:130–135; Littbrand et al., *Phys*

Ther 2006;86:489–498; Conradsson et al., *Aging Ment Health* 2013;17:638–645). However, individuals with dementia may not always be expected to fully understand the overall purpose and scope of the intervention or study. In cases where the participant expresses willingness to participate, consultation must also take place with the participant's next of kin, legal guardian, or trustee, if the matter falls within their mandate.

The individual will not be included in the study if they express, in any form, a desire not to participate, or if any of the consulted parties object to their participation. The procedure for obtaining consent will follow the same approach as in the study titled *"Does physical training reduce dependency in daily activities and the number of falls among older people with dementia? A randomized study"* (Ethical approval no. 2011-205-31M).

The study is expected to provide direct benefit to the participant and contribute to findings that may benefit others with the same or similar condition. The research is also expected to involve minimal risk of harm and minimal discomfort for the participant.

5 Ethical considerations – potential risks

5:1 Describe all potential risks associated with participation in the study.
These may include, for example, physical or psychological harm, pain, discomfort, or breaches of privacy, either short- or long-term. Specify what measures have been taken to prevent the aforementioned risks and what preparedness is in place to manage such complications. Also, indicate which methods will be used to detect, document, and report adverse events.

The intervention will require participants with dementia to travel from their homes to the outpatient unit at the Geriatric Centre twice per week. Adhering to schedules, travelling, changing environments, and meeting new people may cause discomfort such as anxiety, confusion, or distress. Project staff are experienced in working with individuals with cognitive and physical impairments and will be attentive to any signs of discomfort. If a participant experiences distress related to travelling to the Geriatric Centre, home-based interventions may be offered as an alternative. Participants may withdraw from the study at any time without providing a reason.

The intervention includes high-intensity functional exercise, which may carry a risk of physical injury, pain, or other discomforts. However, clinical experience and previous research support the feasibility of physical training in this population, with no serious adverse events reported. The Geriatrics Unit has previously evaluated the same exercise programme in two research projects involving individuals with dementia in residential care settings (Littbrand et al., *Phys Ther* 2006;86:489–498; Toots et al., accepted in *Journal of the American Geriatrics Society*). These studies reported no serious adverse events, and participants with dementia were able to complete the training similarly to those without dementia in terms of attendance, intensity, and discomfort (Littbrand et al., *Phys Ther* 2006;86:489–498).

To minimise risks, the training will be individually tailored and led by physiotherapists experienced in high-intensity training for older adults with cognitive and physical impairments. Each participant's responsible physician will conduct a medical assessment to determine their suitability for participation. Any discomfort experienced during training sessions will be documented using protocols previously applied in two research projects at the unit (FOPANU and UMDEX studies).

Data collection may also be demanding for participants with dementia. Therefore, assessors will be instructed to monitor for signs of fatigue or discomfort. If such signs are observed, the assessor will

consider discontinuing the assessment and will remind the participant of their right to withdraw at any time.

Professor Yngve Gustafson, Chief Physician at the Geriatric Centre, holds medical responsibility for this study, ensuring the safety and care of all participants.

5.2 Describe the potential benefits for the research participants involved in the project (particularly relevant for interventional or treatment research).

Unlike other neurodegenerative diseases such as Multiple Sclerosis (MS) and Parkinson's disease, it is currently uncommon for regional health authorities and municipalities to offer rehabilitation periods specifically for individuals with dementia. This study is therefore expected to address an unmet need among people with dementia and their informal caregivers, and to provide direct benefits to participants allocated to the intervention group.

The intervention is anticipated to have positive effects on several consequences of dementia, such as decline in activities of daily living (ADL) and physical functioning, the presence of behavioural and psychological symptoms of dementia (BPSD), social isolation, depression, and caregiver burden. Improvements in these areas may enhance quality of life and potentially delay the transition to residential care.

Participants allocated to the control group will undergo various assessments and complete questionnaires. They will be offered feedback on their test results. These results will be reviewed by the study's medically responsible physician, and if any serious findings are identified, the participant will be offered the option to have the responsible physician at their primary care centre or the outpatient unit at the Geriatric Centre informed.

The expected benefits for participants are considered to significantly outweigh the potential risks associated with participation in the study.

5.3 Identify and specify any potential ethical issues (benefits/disadvantages) that may arise in a broader perspective as a result of the project.
This may include, for example, whether certain groups (other than the research participants included in the study) might be singled out or receive support as a consequence of the study.

In Sweden, approximately 160,000 individuals are currently living with dementia. Including their relatives and friends, nearly one million people are affected. Dementia can cause significant suffering, as it impacts all aspects of life for both the individual and their informal caregivers. Given the projected dramatic increase in the number of people with dementia, the World Health Organization (WHO) has recommended that dementia be prioritised as a global public health concern.

The negative consequences of dementia are numerous and complex, highlighting the urgent need to develop effective interventions. Despite this, individuals with cognitive impairment are often excluded from research studies. Such exclusion may be considered ethically questionable, as it prevents people with dementia from accessing and evaluating interventions that have proven effective in other patient groups.

The complex nature of dementia indicates a need for person-centred, multidimensional, interdisciplinary rehabilitation to achieve optimal outcomes—namely, to restore or maintain the best

possible functional ability and to promote independence and participation in society. However, such rehabilitation approaches have been scarcely studied in people with dementia. Moreover, unlike other neurodegenerative diseases such as Multiple Sclerosis (MS) and Parkinson's disease, it remains uncommon for regional health authorities and municipalities to offer rehabilitation periods specifically for individuals with dementia.

While the symptoms and progressive nature of dementia may pose challenges to achieving positive outcomes, previous studies examining the effects of individual interventions—or combinations of a limited number of interventions—have shown promising results in this patient group.

6.1 Reporting of results
How is access to the data guaranteed for the principal investigator and participating researchers (e.g., in commissioned research), and who is responsible for data processing and report writing?

Participating researchers are responsible for data processing and report writing. The work is conducted independently of the funding bodies.

6.2 How will the results be made publicly available?
Will the study be submitted for publication in a scientific journal or published in another manner? Specify in what form the results are planned to be disclosed and the timeline for this.

The rehabilitation programme and the results of the study will be disseminated through scientific publications, as well as presentations at national and international conferences. Additionally, findings will be shared through lectures and presentations aimed at interest groups and associations. The results are also expected to be applicable in both undergraduate and continuing professional education across various healthcare professions.

6.3 How is the research participants' right to privacy ensured when the material is made public or published?
Are results reported at the statistical group level? Describe procedures or methods used for de-identification/anonymization.

The results will be presented at the group level, without reference to specific primary care centres or the outpatient unit at the Geriatric Centre. In the data files used for analysis, all participants will be de-identified—no names or personal identification numbers will be recorded.

7. Disclosure of financial interests and conflicts of interest
The disclosure according to sections 7:1–7:3 aims to clarify all direct or indirect relationships that may affect the researcher's relationship with the research participants (e.g., during information provision, consent, or implementation procedures).
7.1 In commissioned research
State the commissioning party, e.g., a company (in clinical drug trials or testing of other new products), an organization, or an authority.
Not applicable.

7.2 Disclose any financial agreements with the commissioning party or other funders (names, amounts).
In clinical drug trials, reference should be made to the agreement entered into with the healthcare provider. Similar agreements may occur in other commissioned research and should be disclosed in the same manner. Separate agreements with the individual(s) conducting the research should also be

reported. Amounts to be received for the study/compensation to the clinic/researcher, what the compensation is intended to cover, and any amounts paid per research participant should also be specified here.

No financial agreements exist.

7.3 Disclose any personal interests of the principal investigator, lead researcher, and participating researchers.
This includes, for example, stock ownership, employment, consultancy assignments in funding companies, or ownership of companies that may (directly or indirectly) benefit financially from the research.

Not applicable.

8. Signatures
Authorized representative of the applicant principal investigator according to section 1.2.

Location: Umeå
Date:

Signature:
Name (printed): Ann Sörlin
Title: Head of Department

Location: Umeå
Date:

Signature:
Name (printed): Håkan Littbrand
Title: Research Assistant

9. List of Appendices
(See Section 9 in the Application Guide.)
Documents that, where applicable, must be attached—unless the corresponding information is already provided in the application form—are marked with an "x." Please indicate which appendices are submitted with this application.
Submitted with the application.

Submitted with the application	Annex no	Description	Clinical drug trial	Other research
☐	1	Participating research principal investigators and collaborating researchers (contact persons) in studies involving more than one research principal. See section 1.4	x	x
☒	2	Research plan intended for pofessionals, and if necessary, an additional appendix intended for laypersons. See section 2.1 and the Guidance for Research Plan/Protocol (Program)	x	x
☐	3	Advertisement materials for the recruitment of research participants. See section 3.1 and the Application Guide,	x	x

		section 3.1."		
☒	4	Written information for those invited to participate. See section 4.1 and the Guidance for Research Participant Information and, where applicable, a separate consent form.	x	x
☒	5	Questionnaire, survey form. See section 2.4.	x	x
☐	6	Common EU form (applicable from May 1, 2004), also applies in case of amendments. For further information, see the website of the Medical Products Agency (Läkemedelsverket) www.lakemedelsverket.se	x	
☐	7	Summary of the protocol in Swedish	x	
☐	8	Investigator's Brochure or alternatively Package Leaflet / Summary of Product Characteristics / Investigator's Brochure (IB)	x	
☒	9	Certificate from the Head of Department or equivalent regarding resources for the safety of research participants. See section 1.5 and suggested format for the resource certificate in the Guidance for Application, section 1.5.	x	x
☒	10	CV of the researcher(s) (same as section 1.3) with main responsibility for the conduct of the study, detailing the researcher(s)' qualifications relevant to the study. See Guidance for Application, section 1.3.	x	x
☐	11	Description of compensation for research participants. See section 3.6 and Guidance for Application, section 3.6.	x	x
