# Supplementary material for: An interdisciplinary rehabilitation program for adults with dementia—A randomized controlled pilot trial evaluating social participation, loneliness and mental health
Source: PLoS One. 2026 Mar 24;21(3):e0345518. doi: 10.1371/journal.pone.0345518 (PMC13012523; doi:10.1371/journal.pone.0345518)
Supplement: S3 File — (PDF) [file pone.0345518.s003.pdf]

Beslutad 2013-03-04

ANSÖKAN OM ETIKPRÖVNING AV FORSKNING SOM AVSER MÄNNISKOR

Information till ansökan, *se Vägledning till ansökan (www.epn.se)*
Beroende på vilken forskning som ansökan gäller kommer de uppgifter som efterfrågas att ha olika relevans. Vid ändring av tidigare godkänd ansökan, se *Vägledning till ansökan*.

Till Regionala etikprövningsnämnden i: Umeå

Den regionala etikprövningsnämnd till vars upptagningsområde forskningshuvudmannen hör, se respektive nämnd *(www.epn.se)*.

Avgift inbetald datum: 2015-08-14

Observera att en ansökan aldrig är komplett och därmed kan behandlas förrän blanketten är korrekt ifylld och avgiften är betald.

Projekttitel: Personcentrerad multidimensionell interdisciplinär rehabilitering av äldre personer med demenssjukdom i ordinärt boende inklusive utbildning och stöd till närstående: en randomiserad kontrollerad studie

Ange en beskrivande titel på svenska för lekmän. Titeln ska ej innehålla sekretesskyddad information. Ange också i förekommande fall, t.ex. vid klinisk läkemedelsprövning, projektets identitet, forskningsplanens/protokollets nummer, version, datum. Vid ändring av tidigare godkänd ansökan, se Vägledning till ansökan.

Projektnummer/identitet: Version nummer: EudraCT nr (vid läkemedelsprövning):

Uppgifter som fylls i av den regionala etikprövningsnämnden

Ansökan komplett:	Dnr:
Begäran om ytterligare information (i sak):	Begärd information inkommen:
Beslutsdatum:	Expeditionsdatum:

Ansökan avser (gäller även vid begäran om rådgivande yttrande):

Forskning där endast en forskningshuvudman deltar (5 000 kr) ☒

Forskning där mer än en huvudman deltar (16 000 kr) ☐

Forskning där mer än en forskningshuvudman deltar, men där samtliga

forskningspersoner eller forskningsobjekt har ett omedelbart samband med endast en av forskningshuvudmännen (5 000 kr) ☐

Endast behandling av personuppgifter (5 000 kr) ☐
(När enbart redan befintliga personregister ska användas, t. ex. nationella databaser)

Forskning som gäller klinisk läkemedelsprövning (16 000 kr) ☐

Ändring av tidigare godkänd ansökan enligt 4 § förordning (2003:615) om etikprövning av forskning som avser människor (2 000 kr) ☐

Om nämnden finner att forskningsprojektet inte faller inom etikprövninglagens tillämpningsområde önskas ett rådgivande yttrande. (Se 4a och 4b §§ i förordning 2003:615 och Vägledning till ansökan)

Ja: ☒ Nej: ☐

1. Information om forskningshuvudman m.m.

1:1 Forskningshuvudman (Se p. 1:1 i Vägledning till ansökan)

Ansökan om etikprövning av forskning ska göras av forskningshuvudmannen. *Med forskningshuvudman avses en statlig myndighet eller en fysisk eller juridisk person i vars verksamhet forskningen utförs.*

Namn: Umeå universitet

Adress: 901 87 Umeå

1:2 Behörig företrädare för forskningshuvudmannen

Behörig företrädare är t.ex. prefekt, enhetschef, verksamhetschef. Forskningshuvudmännen bestämmer själva, genom interna arbets- och delegationsordningar eller genom fullmakt, vem som är behörig att företräda forskningshuvudmannen.

Namn: Ann Sörlin Tjänstetitel: Prefekt

Adress: Umeå universitet, Vårdvetarhuset, 901 87 Umeå

1:3 Forskare som är huvudansvarig för genomförandet av projektet (kontaktperson)

(Se p. 9 bil. nr 10 och p. 1:3 i Vägledning till ansökan)

Observera! Den som är huvudansvarig forskare ansvarar för att andra medverkande som ska genomföra projektet har tillräcklig kompetens (vetenskaplig och klinisk) och vid läkemedelsprövning har tillräcklig kunskap om ”Good Clinical Practice” (GCP). Vid doktorandstudier är som regel handledaren huvudansvarig forskare.

Namn: Håkan Littbrand Tjänstetitel: FoAss

Postadress: Umeå universitet, enheten för Geriatrik, 901 87 Umeå

E-postadress: hakan.littbrand@umu.se

Telefon: 090-785 87 12

Mobiltelefon: 0730-444 851

1:4 Andra medverkande (Se p. 9 bil. nr 1 och p. 1:4 i Vägledning till ansökan)

Övriga deltagande forskningshuvudmän samt forskare ansvariga för att lokalt genomföra projektet (kontaktpersoner) anges här eller i bilaga med namn och adresser (se p. 9 bil. nr 1).

Yngve Gustafson, Professor och överläkare. Enhetschef vid Institutionen för samhällsmedicin och rehabilitering, enheten för Geriatrik, Umeå universitet.

Mia Conradsson, Postdoktor och leg sjukgymnast. Institutionen för samhällsmedicin och rehabilitering, enheten för Geriatrik, Umeå universitet.

Peter Nordström, Professor och överläkare. Institutionen för samhällsmedicin och rehabilitering, enheten för Geriatrik, Umeå universitet.

Maine Carlsson, Medicine doktor och dietist. Geriatriskt centrum, Norrlands universitetssjukhus

Birgitta Olofsson, Docent och leg sjuksköterska. Institutionen för omvårdnad, Umeå universitet.

Nina Lindelöf, Postdoktor och leg sjukgymnast. Institutionen för samhällsmedicin och rehabilitering, enheten för Fysioterapi, Umeå universitet.

Ingeborg Nilsson, Docent och leg arbetsterapeut. Institutionen för samhällsmedicin och rehabilitering, enheten för Arbetsterapi, Umeå universitet.

1:5 Redovisa tillgång till nödvändiga resurser under projektets genomförande

(Se p. 9 bil. nr 9 och p. 1:5 i Vägledning till ansökan)

Ange vem/vilka som har ansvaret (prefekt, verksamhetschef eller motsvarande) för forskningspersonernas säkerhet vid alla enheter/kliniker där forskningspersoner ska delta. Intyg från dessa ansvariga *ska* bifogas (se p. 9 bil. nr 9). Av intyget ska framgå att erforderliga ekonomiska, strukturella och personella resurser finns tillgängliga för att garantera forskningspersonernas säkerhet.

Ann Sörlin, Prefekt vid Institutionen för samhällsmedicin och rehabilitering, Umeå universitet (Bilaga 9a).

Undis Englund, Verksamhetschef vid Geriatriskt centrum, Norrlands universitetssjukhus (Bilaga 9b).

1:6 Ansökan/anmälan till andra myndigheter i vissa fall

(se p. 1:6 i Vägledning till ansökan)	**Insänd**	**Datum**
a)Vid klinisk läkemedelsprövning: Läkemedelsverket	☐	

b)Vid inrättande av biobank: Socialstyrelsen ☐

c)Vid undersökning omfattande joniserande strålning: Strålskyddskommitté ☐

2. Uppgifter om projektet

2:1 Sammanfattande beskrivning av forskningsprojektet

(Se p. 9 bil. nr 2 och p. 2:1 i Vägledning till ansökan)

Beskrivningen ska kunna förstås av nämndens samtliga ledamöter. Undvik därför terminologi som kräver specialkunskaper. Ange bakgrund och syfte för studien samt den/de vetenskapliga frågeställning (ar) som man söker svar på. Ange de viktigaste undersökningsvariablerna. Beskriv vilka kunskapsvinster projektet kan förväntas ge och betydelsen av dessa. Ange om det är en registerstudie, uppdragsforskning etc. För fackmän avsedd detaljerad information om forskningsplan/protokoll (program) *ska* bifogas som bilaga (se p. 9 bil. nr 2). För utformning av forskningsplan/protokoll se p. 2:1 i Vägledning till ansökan. Ange när datainsamlingen beräknas vara avslutad. En utförligare beskrivning av studiens genomförande *avsedd för lekmän* kan vid behov bifogas den för fackmän avsedda obligatoriska forskningsplanen.

Bakgrund:

I Sverige finns idag cirka 160 00 personer med demenssjukdom och inkluderat närstående och vänner påverkas sammanlagt cirka en miljon människor. Demens kan innebära ett stort lidande då det är en sjukdom som påverkar hela livssituationen för de som drabbas och för deras närstående. Demenssjukdom har ett progressivt förlopp och är den främsta orsaken till beroende i aktiviteter i dagligt liv (ADL) bland äldre personer. Inom 2-3 år har cirka hälften av de som fått diagnos flyttat till ett särskilt boende.

Demens ger en gradvis försämrad kognitiv funktion, vilket innefattar bland annat minne, uppmärksamhet, inlärning, och problemlösning. Förutom detta kan konsekvenser av demenssjukdomen vara många och komplexa. Nedsatt balans och gångförmåga är vanligt, vilket tillsammans med nedsatt kognition ger ökad risk för fall och fallskador. Nedsatt förflyttningsförmåga, tillsammans med svårigheter att orientera sig och nedsatt initiativförmåga, gör att personen med demens även riskerar att ha få sociala kontakter och låg fysisk aktivitetsnivå. Studier har visat att man kan påverka ADL-förmåga, kognitiv funktion och välbefinnande bland personer med demens genom interventioner med fysisk eller social aktivitet. Detta indikerar möjlighet att påverka förloppet och betydelsen av att bibehålla dessa funktioner så länge som möjligt. Andra aspekter på den komplexa problematiken är att personer med demens kan ha en reducerad fysiologisk reservkapacitet vilket innebär en ökad risk för att drabbas av

delirium av interna och externa störningar, som till exempel smärta eller faktorer i miljön, samt större risk att drabbas av komplikationer vid sjukdom eller läkemedelsrelaterade problem. Vidare löper personer med demens också en ökad risk för andra medicinska tillstånd såsom malnutrition, inkontinens, nedsatt munhälsa, samt depression. Utöver depression är symtom såsom ångest, aggression, rastlöshet, hallucinationer, vandringsbeteende och sömnstörningar vanligt. Dessa symtom benämns ofta som beteendemässiga och psykiska symtom vid demens, BPSD. BPSD ger stort lidande för personen med demens, men även för närstående, och är den vanligaste orsaken till att personen med demens flyttar till särskilt boende. BPSD, kognitiv nedsättning och behov av fysisk hjälp i vardagen påverkar omfattningen av insatserna från en vårdande närstående, och kan ha negativ påverkan på den närståendes psykiska och fysiska hälsa, livskvalité, ekonomi och deltagande i samhället.

Många av komplikationerna relaterade till demenssjukdomen är möjliga att förhindra och påverka. Den komplexa problematiken vid demenssjukdom indikerar behov av personcentrerad multidimensionell interdisciplinär rehabilitering för att personen med demens ska ha goda möjligheter att återvinna eller bibehålla bästa möjliga funktionsförmåga samt skapa goda villkor för ett självständigt liv och aktivt deltagande i samhället. Personcentrerad multidimensionell interdisciplinär rehabilitering innebär en process där ett team, bestående av många olika yrkesprofessioner, gör en utförlig bedömning av individen för att identifiera problem och behov samt styrkor och resurser. Teamet, tillsammans med personen med demens och närstående, kommer därefter överens om åtgärder och målsättning med rehabiliteringsperioden. Hur rehabilitering framåtskrider utvärderas sedan på regelbundna möten. Detta arbetssätt används idag kliniskt inom geriatrisk rehabilitering. Både sammanställningar av Statens beredning för medicinsk utvärdering (SBU) och metaanalyser av The Cochrane Collaboration har kommit fram till att arbetssättet är framgångsrikt vid vård och rehabilitering av äldre patienter med komplexa problem i slutenvård. Bland annat fann man positiva effekter på ADL-förmåga och på möjligheterna att bo kvar i ordinärt boende efter sjukhusvistelsen. Arbetssättet är dock inte utvärderat specifikt bland personer med demens i öppenvård.

Ett viktigt komplement till rehabiliteringen av personer med demenssjukdom är att erbjuda närstående utbildning samt stöd och rådgivning kring problem som härrör från sjukdomen för att kunna hantera det vardagliga livet bättre. Studier har visat att denna typ av insats kan var viktig för att kunna minska den upplevda belastningen, depressionssymtom och negativ påverkan på hälsa för den vårdande närstående samt för att kunna förlänga tiden som personen med demens kan bo kvar i ordinärt boende.

Syfte och genomförande

Forskningsprojektet kommer att utvärdera effekter av ett personcentrerat multidimensionellt interdisciplinärt rehabiliteringsprogram för personer med demenssjukdom som bor i ordinärt boende, inkluderat utbildning, stöd och rådgivning till vårdande närstående. Forskningspersonerna lottas till sedvanlig vård eller till ett personcentrerat multidimensionellt interdisciplinärt rehabiliteringsprogram under 16 veckor inklusive två uppföljningstillfällen (fem och femton månader efter avslutad rehabiliteringsperiod). Den primära frågeställningen är om detta program kan motverka konsekvenser av demenssjukdomen och därmed möjliggöra att en större andel personer med demens bor kvar i ordinärt boende 2 och 3 år efter påbörjad intervention.

Sekundära frågeställningar är huruvida programmet:

-minskar upplevelsen av belastning för närstående som vårdar en anhörig med demenssjukdom samt ökar hälsorelaterad livskvalité och motverkar depression.

-ökar delaktighet i samhället, motverkar depression och nedsatt välbefinnande bland personer med demens.

-motverkar/bromsar följder av demenssjukdom såsom fysisk inaktivitet, nedsättning i kognitiv och fysisk funktionsnivå, ökat hjälpbehov i aktiviteter i dagliga livet, förekomst av beteendemässiga och psykiska symtom vid demens (BPSD), fall och fallskador, malnutrition, samt minskar användning av olämpliga läkemedel hos personer med demens.

-är kostnadseffektivt.

-är genomförbart utifrån hur det upplevs att delta för personer med demens och deras närstående, samt utifrån närvaro och eventuella biverkningar/obehag relaterade till interventionen.

-skiljer sig i genomförande och effekt utifrån kön, ålder, tillgång till vårdande närstående, kognitiv funktion, och förekomst av BPSD.

Datainsamlingen beräknas vara avslutad i november 2020 (se mer i detalj i forskningsplan, Bilaga 2).

Betydelse:

Projektets övergripande mål är att utveckla och förbättra vården för personer med demenssjukdom. Det är idag ovanligt, till skillnad från andra neurodegenerativa sjukdomar som till exempel Multipel Skleros (MS) och Parkinsons sjukdom, att landsting och kommuner erbjuder rehabiliteringsperioder specifikt för demenssjukdom. Demenssjukdom är en av de mest

resurskrävande av kroniska sjukdomar och den årliga kostnaden i Sverige har beräknats till cirka 63 miljarder kronor. Eftersom antalet personer med demens kommer att öka dramatiskt i framtiden förordar WHO att demens bör prioriteras som ett globalt folkhälsoproblem. Den komplexa problematiken vid demenssjukdom indikerar ett behov av personcentrerad multidimensionell interdisciplinär rehabilitering för att uppnå optimal effekt, det vill säga att återskapa eller bibehålla bästa möjliga funktionella förmåga, samt skapa förutsättningar för oberoende och deltagande i samhället. Det vetenskapliga underlaget för denna typ av rehabilitering bland personer med demens är i dagsläget begränsat. Interventionen förväntas ha positiva effekter på konsekvenser av demenssjukdomen som till exempel försämring i ADL och fysisk funktionsförmåga, förekomst av BPSD, social isolering, depression och närståendebelastning, vilket kan påverka möjligheten att bo kvar i ordinärt boende. Positiva effekter av programmet skulle kunna ha stor klinisk betydelse då det kan vara en metod som tillhandahålls kliniker för att motverka lidande som följd av demenssjukdom. Vid fördröjning av flytt till särskilt boende kan programmet även leda till stora besparingar för samhället.

Se även bifogad forskningsplan (Bilaga 2).

2:2 Vilken/vilka vetenskaplig (a) frågeställning (ar) ligger till grund för projektets utformning?

Om projektet kan karakteriseras som en hypotesprövning, ange den primära och eventuellt sekundära hypotesen. Hänvisning till mer detaljerad information för fackmän kan ske till bifogad forskningsplan/protokoll enligt punkt 2:1.

Den primära hypotesen är att ett personcentrerat multidimensionellt interdisciplinärt rehabiliteringsprogram för personer med demenssjukdom i ordinärt boende, inklusive utbildning, stöd och rådgivning till vårdande närstående, ökar andelen som lever och bor kvar i ordinärt boende efter två år (primär utfallsvariabel).

Sekundära hypoteser är att programmet:

-minskar upplevelsen av belastning för närstående som vårdar en anhörig med demenssjukdom samt ökar hälsorelaterad livskvalité och motverkar depression.

-ökar delaktighet i samhället, motverkar depression och nedsatt välbefinnande bland personer med demens.

-motverkar/bromsar följder av demenssjukdom såsom fysisk inaktivitet, nedsättning i kognitiv och fysisk funktionsnivå, ökat hjälpbehov i aktiviteter i dagliga livet, förekomst av

beteendemässiga och psykiska symtom relaterade till demenssjukdom, fall och fallskador, malnutrition, samt minskar användning av olämpliga läkemedel hos personer med demens.

-är kostnadseffektivt.

-är genomförbart för personer med demens och deras närstående utifrån en positiv upplevelse att delta, hög närvaro och att inga allvarliga biverkningar/obehag inträffar relaterade till interventionen.

2:3 Redogör för resultat från relevanta djurförsök (Gäller klinisk behandlingsforskning)

Om djurförsök inte utförts ange skälen till detta.

Djurförsök ej relevant.

2:4 Redogör översiktligt för undersökningsprocedur, datainsamling och datas karaktär

(Se p. 9 bil. nr 5 och p. 2:4 i Vägledning till ansökan)

Av beskrivningen ska framgå hur projektet planeras genomföras. Beskriv insamlade datas karaktär. Ange hur datas tillförlitlighet säkerställs (t.ex. kvalitetskontroll/monitorering). Vid enkäter och intervjuer ska beskrivas tillvägagångssätt och t.ex. frågors innehåll och hur slutsatser dras. Enkäter och skattningsskalor *ska* bifogas (se p. 9 bil. nr 5). För medicinsk forskning ska anges t.ex. typer av ingrepp, mätmetoder, antal besök, tidsåtgång vid varje försök, doser och administrationssätt för eventuella läkemedel och/eller isotoper, blodprovsmängd (även ackumulerad mängd vid multipla försök). Ange om och på vilket sätt undersökningsprocedur m.m. skiljer sig från klinisk rutin. Om en behandling studeras för första gången på människa ska detta framgå och relevanta säkerhetsrutiner beskrivas. Ange proceduren för att ge den eventuella behandling efter projektets slut, som kan erfordras. Ange procedur för insamling av biologiskt material. Redogör för datakällor och procedurer vid behandling av personuppgifter. För mer detaljerad information kan hänvisning ske till bilagd forskningsplan.

Intervention

Forskningspersonerna lottas till en av två grupper, kontroll eller intervention, efter att baslinjemätningar är avklarade. Att lottas till kontrollgrupp innebär att forskningspersonerna får sedvanlig/ordinarie vård och stöd. Intervention innebär att forskningspersonerna med demenssjukdom skrivs in som patienter vid öppenvårdsenheten, Geriatriskt centrum, Norrlands universitetssjukhus. Interventionen erbjuds i lokaler vid Geriatriskt centrum som är lämpliga för dagrehabiliterande verksamhet, men även insatser i hemmet eller utanför hemmet kan bli aktuellt beroende av de individuella rehabiliteringsmålen. Ett interdisciplinärt team bestående av läkare, sjuksköterska, undersköterska, sjukgymnast, arbetaterapeut, kurator, dietist, neuropsykolog, tandhygienist och apotekare, vilka har erfarenhet av rehabilitering av äldre personer med nedsatt kognitiv och fysisk förmåga, samarbetar i bedömning och behandling av forskningspersonen. Varje försöksperson bedöms enligt följande möjliga problemområden vid demenssjukdom: funktionell fysisk kapacitet, kognitiv funktion, ADL-förmåga, fallrisk, deltagande i samhället,

fysisk aktivitet, nutrition, sjukdomar, beteendemässiga och psykiska symtom, samt läkemedel. Baserat på identifierade problemområden, men även styrkor, resurser och egna önskemål, bildas ett team runt varje forskningsperson med demens. Teamet kommer överens om åtgärder och individuella rehabiliteringsmål. Kuratorn bedömer behov av individuellt stöd och rådgivning för närstående. Efter interventionsperioden på 16 veckor sker överrapportering till hälsocentralen och det medicinska ansvaret av forskningspersonen med demenssjukdom återtas av hälsocentralen.

Personen med demens erbjuds följande insatser under interventionsperioden på 16 veckor:

1. Fysisk aktivitet: individanpassad funktionell träning under 45 minuter, 2 ggr/vecka, med sjukgymnast, med målsättning att förbättra muskelstyrka, balans och gång- och förflyttningsförmåga. Träningen är baserad på High-Intensity Functional Exercise Program (HIFE Program). Individuella råd ges för att säkerställa fysisk aktivitet med minst moderat intensitet under 150 minuter/vecka, enligt rådande hälsofrämjande rekommendationer.

2. Individuella målinriktade åtgärder: upp till två sessioner per vecka baserat på individuella mål för rehabiliteringsperioden, utfört av lämplig profession i teamet. Insatser som erbjuds grundar sig på evidens och beprövad klinisk erfarenhet.

Närstående erbjuds följande insatser under interventionsperioden på 16 veckor:

1. Gruppträffar: vid sex tillfällen erbjuds närstående att delta i gruppträffar bestående av information och diskussion utifrån ett specifikt tema, där temat planeras utifrån/tar hänsyn till deltagarnas behov och önskningar. Teman för sessioner kan exempelvis vara demenssjukdom och dess förlopp, hur man kan hantera och förebygga BPSD, hälsofrämjande aktiviteter eller samhällsservice. En kurator inhämtar deltagande närståendes behov och önskemål inför interventionsperioden, samt är en av gruppledarna under sessionerna. Gruppledaren verkar också för att stimulera social interaktion mellan närstående som deltar i studien.

2. Stöd och rådgivning vid behov: närstående erbjuds samtal med kurator upp till sex gånger under interventionsperioden. Råd och stöd kan ges exempelvis angående möjlighet och struktur för formell vård, ekonomiskt stöd, psykologiskt stöd samt hantering av BPSD.

Uppföljningar

Efter rehabiliteringsperioden kommer interventionen att följas upp vid två tillfällen, fem och femton månader efter interventionsperiodens slut. Personen med demens kommer att följas upp av teamet avseende hur det gått att uppfylla de hälsofrämjande rekommendationerna rörande

fysisk aktivitet samt de individuella rehabiliteringsmålen utifrån genomförda insatser under rehabiliteringsperioden. Vid behov kommer insatserna modifieras. För närstående kommer uppföljningen att ske av kurator. Kurator kommer att vid behov ge råd och vägledning.

Datainsamling

Forskningspersonerna i interventions- och kontrollgrupp kommer genomgå testning vid inklusion i studien, dvs baslinjen, samt efter 4 (avslutad rehabiliteringsperiod), 12, 24, och 36 månader. Testning innefattar bedömningar bestående av skattningsskalor och intervjufrågor, och sker vid ett hembesök av en person som är blind för vilken grupp försökspersonen tillhör. Besöket hos forskningspersonen med demens förväntas ta cirka 2 timmar, och kan delas upp på två tillfällen om behov finns. Besöket hos närstående kommer att ta cirka 1 timme. I tillägg kommer en del information om forskningspersonen med demens samlas in från patientjournaler. De bedömningsinstrument och skattningsskalor som används är reliabla och valida. De som används för personen med demens är tillämpbara för personer med nedsatt kognitiv förmåga. Testning genomförs av personal med utbildning för denna typ av bedömningar och som är blindade för vilken grupp forskningspersonen tillhör.

Huvudutfall

Datum för eventuell flytt till särskilt boende kommer att samlas in genom journaler i kommunen. Eventuella dödsdatum samlas in genom landstingets medicinska journaler.

Delutfall

Närstående:

Närståendebelastning kommer att samlas in genom att använda Anhörigbördaskalan. Skalan innehåller frågor till den närstående inom fem dimensioner: allmän belastning, isolering, besvikelse, emotionell påverkan och miljö. Depressiva symtom samlas in med Geriatrisk depressionsskala (GDS-15) och hälsorelaterad livskvalité med SF-36.

Person med demens:

Depressiva symtom mäts med Geriatrisk depressionsskala (GDS-15). Psykologiskt välbefinnande mäts med Philadelphia Geriatric Center Morale Scale (PGCMS).

Delaktighet i samhället kommer att samlas in genom att fråga närstående alternativt personal rörande antal besök i hemmet, antal besök till andra personer och kontakter med släkt och vänner via telefon eller andra sociala medier.

Fysisk aktivitet kommer att samlas in med IPAQ - E (International Physical Activity Questionnaire –Elderly) som är ett frågeformulär om aktivitetsvanor. Tillägg till frågeformuläret har gjorts av forskargruppen.

Kognitiv funktion mäts med Alzheimer Disease Assessment Scale-Cognitive Subscale (ADAS-Cog), Mini-Mental Test (MMT) och verbalt flöde.

Balans-, gång- och förflyttningsförmåga kommer att mätas med Bergs balansskala, "Chair-stand" (dvs. förmågan att resa sig up från en stol) samt gånghastighet över 2,4 meter.

Självständighet i ADL kommer att mätas med Functional Independence Measure (FIM) och Lawton scales (P- och IADL) genom att fråga personal eller närstående som känner personen med demens väl.

Förekomst av beteendemässiga och psykiska symtom relaterade till demenssjukdom kommer att mätas med Neuropsychiatric Inventory (NPI) genom intervjuer av personal eller närstående som känner forskningspersonen med demens väl.

Näringsstatus kommer att mätas med Mini Nutritional Status (MNA) genom mätningar av forskningspersonen och frågor till personal eller närstående som känner forskningspersonen med demens väl. Body Mass Index (BMI) kommer att registreras genom att mäta längd och vikt.

Olämpliga läkemedel inklusive interaktioner samlas in med utgångspunkt från Socialstyrelsens rekommendationer, http://www.socialstyrelsen.se/Lists/Artikelkatalog/Attachments/18085/2010-6-29.pdf. Läkemedelsanvändning samlas in genom APO-dos registret.

Fall och fallskador följs upp via medicinska journaler och kommunens avvikelserapportering.

Teamet kommer att registrera negativa händelser/obehag under interventionen i protokoll. Närstående eller personal kommer att tillfrågas regelbundet om forskningspersonen uppvisar några obehag relaterat till att åka till, eller i samband med vistelse på Geriatriskt centrum. Vid varje träningstillfälle kommer sjukgymnasten att registrera eventuella negativa eller positiva händelser och reaktioner, motivationsnivå och uppnådd träningsintensitet för varje forskningsperson på protokoll som tidigare använts i träningsstudier av personer med demens av forskargruppen. Upplevelse av interventionen kommer även att beskrivas genom att intervjua 15 forskningspersoner med demens respektive närstående. Inledningsfrågan till närstående kommer att vara: Hur upplever du det att ta del av de insatser du erbjudits från Geriatriskt centrum? En uppföljningsfråga ställs till alla: Är det något du skulle vilja ändra på? Intervjuerna till personen med demens görs i anslutning till ett besök eller en träning/aktivitet i hemmet för att deltagaren lättare ska minnas och kunna relatera frågorna till interventionen. Intervjuerna kan vid behov göras vid två tillfällen. Inledningsfråga kommer att vara: Hur upplever du det att ta del av de

aktiviteter du deltagit i? En uppföljningsfråga ställs till alla: Är det något du skulle vilja ändra på? Intervjuerna analyseras med kvalitativ innehållsanalys.

Kostnadseffektivitet kommer att analyseras med hjälp av EQ-5D och beräkningar av kostnader för intervention, konsumtion av sjukvård och social service. Konsumtion av sjukvård och social service kommer att mätas genom att registrera antal dagar inneliggande på sjukhus och öppenvårdsbesök genom att gå igenom medicinska journaler samt genom att registrera formell och informell vård och omsorg med Resource Utilazation in Dementia (RUD)-instrument.

Sjukdomar och mortalitet kommer att följas under studietiden (36 månader) genom granskning av medicinska journaler.

De datakällor som kommer att användas i studien är forskningspersonens journal i kommun och Västerbottens läns landsting, samt Apo-dos registret.

All data kommer att behandlas konfidentiellt. Varje försöksperson får vid inklusion ett unikt kodnummer. I datafilerna, som används som underlag till analys av resultatet, avidentifieras materialet och endast forskningspersonens kodnummer kommer att registreras, det vill säga inga personnummer kommer att förekomma i datafilerna. Kodlistor förvaras skiljt från inmatade datafiler i brandsäkra utrymmen som endast behöriga forskare har tillgång till. Dokumentering av insatser under interventionen kommer att ske på pappersprotokoll tillhandahållna av projektet. För personen med demens i interventionsgruppen, som är inskrivna vid öppenvårdsenheten vid Geriatrisk centrum, kommer bedömningar och insatser även att dokumenteras i Västerbottens läns landstings datajournal vid Geriatriskt centrum.

Se även bifogad forskningsplan (Bilaga 2) och skattningsskalor/frågeformulär (Bilaga 5)

2:5 Redogör för om insamlat biologiskt material kommer att förvaras i en biobank

(Se p. 2:5 i Vägledning till ansökan)

Med biobank avses biologiskt material från en eller flera människor som samlas och bevaras tills vidare eller för en bestämd tid och vars ursprung kan härledas till den eller de människor från vilka materialet härrör. Redogör för var och hur prover som ska sparas förvaras, kodningsprocedurer och villkor för utlämnande av prover. Ange huvudman för biobanken.

Ej aktuellt.

2:6 Dokumentation, dataskydd och arkivering (Se p. 2:6 i Vägledning till ansökan)

Redogör för hur undersökningsprocedurer och eventuella ingrepp dokumenteras. Ange om band- och videoinspelningar används. Om materialet ska kodas, ange proceduren, vem som förvarar kodlistor/kodnycklar och vem eller vilka som har tillgång till dem, var och hur länge de förvaras samt om materialet kommer att anonymiseras eller förstöras. Redogör för vilken tillgänglighet datamaterialet har och hur det förvaras samt hur erforderligt sekretesskydd erhålls.

Information från testtillfällen dokumenteras i pappersprotokoll och dessa förvaras så att endast personal i projektet har tillgång till dem. Varje forskningsperson får vid inklusion ett unikt kodnummer. Efter datainsamling matas data in i statistikprogram, och ljudfiler från intervjuer transkriberas till textfiler. Inga videoinspelningar kommer att användas vid undersökningen. I datafilerna, som används som underlag till analys av resultatet, avidentifieras materialet och endast forskningspersonens kodnummer kommer att registreras, det vill säga inga personnummer kommer att förekomma i datafilerna. Vid transkriberingen sker en avidentifiering av deltagarna. Kvalitativ innehållsanalys är en etablerad forskningsmetod som kommer att användas för att analysera intervjuerna (kvalitativ data). Kodlistor, pappersprotokoll och ljudfiler förvaras skilt från inmatade datafiler och arkiveras i ett brandsäkert låst utrymme dit endast behöriga forskare har tillträde. De elektroniska datafilerna och textfilerna backas upp på server tillhandahållen av Umeå universitet. Datum när de elektroniska filerna skapas registreras.
Allt forskningsmaterial kommer att sparas minst 10 år efter sista vetenskapliga publikation från projektet.

2:7 Redogör för tidigare erfarenheter (egna och/eller andras) av den använda proceduren, tekniken eller behandlingen

Särskilt angeläget är att redovisning av risker för komplikationer görs tydliga och i förekommande fall med angivande av relevanta publikationer. Vid nya behandlingar av patienter, t.ex. med läkemedel, bör anges hur många patienter (med aktuell eller annan åkomma) som tidigare erhållit föreslagen behandling, läkemedelsdosering (eller annan dosering) samt hur långa behandlingsperioder som studerats.

Studien kommer att genomföras av forskare vid Umeå universitet, Institutionen för samhällsmedicin och rehabilitering, enheterna för Geriatrik, Fysioterapi och Arbetsterapi, samt Institutionen för omvårdnad. Enheterna har mångårig erfarenhet av att genomföra och analysera resultat från interventionsstudier bland sköra äldre personer med kognitiva och fysiska nedsättningar (Jensen et al. Ann Intern Med 2002;136:733-741, Rosendahl et al. Aust j Physiother 2006;52:105-113, Littbrand et al. J Am Geriatri 2009;57:1741-1749, Lundström M et

al. J Am Geriatri 2005;53:622-628). Forskargruppen har även stor erfarenhet, både inom klinik och forskning (Lundström M et al. J Am Geriatri 2005;53:622-628, Stenvall et al. Arch Gerontol Geriatr 2012;54:e284-289, Jensen et al. Ann Intern Med 2002;136:733-741), att arbeta med interdisciplinärt teamarbete. Träningsprogrammet (HIFE programmet) som kommer att användas i studien har forskargruppen själva utvecklat med utgångspunkt att det ska vara genomförbart bland personer med nedsatt kognitiv och fysisk förmåga. HIFE programmet har utvärderats bland personer på särskilt boende inklusive personer med demens (Littbrand et al. Phys Ther 2006;86:489-498). Inga allvarliga biverkningar med bestående skada eller sjukdom relaterade till träningsprogrammet har förekommit. Deltagare med demens har kunnat genomföra träningen på liknande sätt som de utan demens, avseende närvaro, träningsintensitet och obehag.

3. Uppgifter om forskningspersoner

3:1 Hur görs urvalet av forskningspersoner? (Se p. 9 bil. nr 3 och p. 3:1 i Vägledning till ansökan)

Med forskningsperson avses en levande människa som forskningen avser. Ange urvalskriterier (inklusion och exklusion). Redogör för på vilket sätt forskaren kommer i kontakt med/får kännedom om lämpliga forskningspersoner. Om annonsering sker, *ska* annonsmaterialet insändas som bilaga (se p. 9 bil. nr 3). Om t.ex. barn eller personer som tillfälligt eller permanent inte är kapabla att ge ett eget informerat samtycke ska ingå i projektet, ska detta särskilt motiveras. Om vissa grupper utesluts från deltagande i projektet ska detta särskilt motiveras.

Studien kommer att genomföras vid Geriatriskt centrum, Norrlands universitetssjukhus i Umeå, och deltagare rekryteras från Umeå kommun med omgivande kranskommuner. Inklusionskriterier för forskningspersonerna med demens innefattar: diagnostiserad demensdiagnos, 60 år eller äldre, boende i ordinärt boende, 10 poäng eller mer på Mini-Mental Test (MMT), tillstyrkande från ansvarig läkare att delta i studien, flytt till särskilt boende är inte initierad (det vill säga står ej i kommunens kösystem för flytt till särskilt boende eller innehar växelvård), förväntad överlevnad är mer än sex månader, förmåga att ställa sig upp självständigt eller med stöd av armstöd och/eller en person, samt tillräckligt god hörsel och svenska språkkunskaper som möjliggör att forskningspersonen kan delta i mätningar. Inklusionskriterier för närstående innefattar: person som har ansvar för vård och stöd av forskningspersonen med demenssjukdom. Maximalt två personer med ansvar kommer erbjudas att vara med i studien för respektive forskningsperson med demenssjukdom. Med närstående menas både personer inom familjen eller släkten och andra personer som till exempel grannar eller vänner som hjälper den sjuka.

Deltagare rekryteras via hälsocentraler och mottagningen vid Geriatriskt centrum, Norrlands universitets sjukhus. Utifrån riktlinjer från projektet identifieras potentiella forskningspersoner av

sköterska eller läkare vid hälsocentraler (företrädesvis av demensansvarig person) eller mottagningen vid Geriatriskt centrum. De tar den första kontakten och frågar den möjlige deltagaren om forskare från projektet får ta kontakt med anledning av studien. Skriftlig information skickas till den möjliga deltagaren och därefter ges även muntlig information. Information och förfrågan till närstående om intresse att delta går ut i samband med att deltagaren med demens inkluderas. Intresseförfrågan om att vara med i studien kommer även att utgå genom Demensförbundets lokala föreningar i Umeå med kranskommuner. Personer som är intresserade av att vara med ombeds att ta kontakt med forskare i projektet (se informationsbrev bilaga 4b). Ingen annonsering kommer att ske för att rekrytera forskningspersoner.

Klinisk erfarenhet och erfarenhet från egen och andras forskning stödjer att personer med demenssjukdom som har en kognitiv funktion motsvarande minst 10 poäng på Mini-Mental Test kan uttrycka sitt intresse att deltaga i en aktivitet och svara tillförlitligt på sin upplevelse av testning eller träning (Mowley et al. Int J Geriatr Psychiatry 1999; 14:776-783, Hoe et al. Age Ageing 2005; 34:130-135, Littbrand et al. Phys Ther 2006; 86:489-498. Conradsson et al. Aging Ment Health 2013; 17:638-645) Forskningspersoner med demenssjukdom kan dock inte alltid fullt ut förväntas att förstå vad intervention/studien innebär i sin helhet. I de fall forskningspersonen är positiv till att delta i studien skall även samråd ske med forskningspersonens närmaste anhöriga eller i samråd med god man eller förvaltare om frågan ingår i dennes uppdrag. Studien förväntas leda till direkt nytta för forskningspersonen samt bidra till ett resultat som kan vara till nytta för forskningspersonen eller någon annan som lider av samma eller liknande sjukdom eller störning. Forskningen förväntas även innebära en obetydlig risk för skada och ett obetydligt obehag för forskningspersonen.

3:2 Ange relationen mellan forskare/försöksledare och forskningspersonerna

☒ Behandlare (t.ex. läkare, psykolog, sjukgymnast) - forskningsperson (t.ex. patient, klient)

☐ Kursgivare (lärare) - student

☐ Arbetsgivare - anställd

☐ Annan relation som kan tänkas medföra risk för påverkan. Beskriv:

3:3 Redogör för det statistiska underlaget för studiepopulationens (-ernas)/ undersökningsmaterialets (-ens) storlek (Se p. 3:3 i Vägledning till ansökan)

Redovisa statistisk styrka, så kallad ”power”- beräkning eller redovisa motsvarande överväganden som tydliggör studiens möjligheter att besvara frågeställningarna.

Inom 2-3 år har cirka hälften av de som fått diagnos flyttat till ett särskilt boende (SBU – the Swedish Council on Technology Assessment in Health Care. Dementia – Etiology and

Epidemiology. 2008). En poweranalys (80%, P=0.05, 2-sidig) är gjord baserad på en förväntad skillnad mellan interventions- och kontrollgrupp vid 24-månaders uppföljning. Den förväntade skillnaden är 50% i andel deltagare som lever och bor i ordinärt boende (75% i interventionsgrupp och 50% i kontrollgrupp), vilket är det omvända förhållandet vid en tidpunkt till död eller flytt till särskilt boende kombinerat. Analysen visar ett behov av 179 deltagare med demenssjukdom, inkluderat ett beräknat bortfall under studiens gång på 5%.

3:4 Kan forskningspersonerna komma att inkluderas i flera studier samtidigt eller i nära anslutning till denna? I så fall, vilken typ av forskning?

(Se p. 3:4 i Vägledning till ansökan)

Forskningspersonerna kommer inte att inkluderas i fler studier som i dagsläget är känt.

3:5 Vilket försäkringsskydd finns för de forskningspersoner som deltar i projektet?

Det åligger forskningshuvudmannen att kontrollera att det finns försäkring som täcker eventuella skador som kan uppkomma i samband med forskningen.

Forskningspersonerna med demenssjukdom omfattas av patientförsäkringen då de blir inskrivna vid Geriatriskt centrum, Norrlands universitetssjukhus. För närstående som inkluderas i studien har ingen särskild försäkring tecknats då de inte erhåller någon behandling.

3:6 Vilken ekonomisk ersättning eller andra förmåner utgår till de forskningspersoner som deltar i projektet och när betalas ersättningen ut? Utförligare beskrivning kan lämnas i bilaga.

(Se p. 9 bil. nr 11 och p. 3:6 i Vägledning till ansökan)

Ersättning för obehag och besvär. Ange belopp (före skatt):

Ersättning för förlorad arbetsinkomst	☐ Ja	☒ Nej
Reseersättning	☒ Ja	☐ Nej
Befrielse från kostnader för läkemedel	☐ Ja	☒ Nej

Befrielse från andra kostnader. Vilka?

Forskningspersonerna med demens får ersättning för patientavgifter.

Andra förmåner. Vilka?

När betalas ersättningen ut?

Patientavgifter och avgifter för sjukresor faktureras direkt till projektet. Reseersättning för inkluderade närstående betalas ut efter inlämnat underlag.

Ingen ersättning betalas ut ☐

4. Information och samtycke

4:1 Proceduren för och innehållet i den *information* som lämnas då forskningspersoner tillfrågas om deltagande

(Se p. 9 bil. nr 4 och Vägledning till forskningspersonsinformation.)

Enligt 16 § lag (2003:460) om etikprövning av forskning som avser människor ska forskningspersonen informeras om den övergripande planen för forskningen, syftet med forskningen, de metoder som kommer att användas, de följder och risker som forskningen kan medföra, vem som är forskningshuvudman, att deltagande i forskningen är frivilligt och forskningspersonernas rätt att när som helst avbryta sin medverkan. Beskriv hur och när information ges och vad den innehåller. Ange vem som informerar. Normalt ska en kortfattad och lättförståelig skriftlig information ges. Denna skriftliga information *ska* bifogas ansökan (se p. 9 bil. nr 4). Om ingen eller ofullständig information ges, måste skälen för detta noggrant anges.

Potentiella forskningspersoner, det vill säga personen med demenssjukdom och vårdande närstående, informeras dels skriftligt (se Bilaga 4a och 4b, respektive Bilaga 4c), dels muntligt (samma innehåll) med möjlighet att ställa frågor. Den skriftliga informationen kommer att skickas till forskningspersonen innan en representant från projektet besöker forskningspersonen och ger informationen muntligt (se även beskrivning rekrytering under punkt 3:1).

4:2 Hur och från vem inhämtas *samtycke*? (Se Vägledning till forskningspersonsinformation)

Beskriv proceduren; vem som frågar, när detta sker och hur samtycket dokumenteras. Utförlig redovisning är särskilt viktig då barn eller personer med nedsatt beslutskompetens ingår i studien, likaså vid studier av en grupp/grupper, t.ex. skolklasser, föreningar, organisationer, företag, kyrkosamfund, församlingar eller grupper som interagerar inom sociala medier.

Informerat muntligt samtycke att delta i projektet efterfrågas från forskningspersonen och dokumenteras av representanter i projektet, efter att både muntlig och skriftlig information givits samt frågor besvarats (se Bilaga 4d). Forskningspersonen kan när som helst avbryta deltagande i studien utan att motivera det. De kan även välja att tacka nej till delar av undersökningen och interventionen såsom till exempel enstaka undersökningar eller aktivitetspass.

Klinisk erfarenhet och erfarenhet från egen och andras forskning stödjer att personer med demenssjukdom som har en kognitiv funktion motsvarande minst 10 poäng på Mini-Mental Test kan uttrycka sitt intresse att deltaga i en aktivitet och svara tillförlitligt på sin upplevelse av testning eller träning (Mowley et al. Int J Geriatr Psychiatry 1999; 14:776-783, Hoe et al. Age Ageing 2005; 34:130-135, Littbrand et al. Phys Ther 2006; 86:489-498. Conradsson et al. Aging Ment Health 2013; 17:638-645). Forskningspersoner med demenssjukdom kan dock inte alltid fullt ut förväntas att förstå vad intervention/studien innebär i sin helhet. I de fall forskningspersonen är positiv till att delta i studien skall även samråd ske med forskningspersonens närmaste anhöriga eller i samråd med god man eller förvaltare om frågan ingår i dennes uppdrag. Den aktuella forskningspersonen kommer inte att inkluderas i studien om

forskningspersonen i någon form ger uttryck för att inte vilja delta eller om någon av personerna som samråd har skett med motsätter sig detta. Proceduren för inhämtning av samtycke sker i huvudsak med samma tillvägagångssätt som i studien med projektiteln "Leder fysisk träning till minskat hjälpberoende i vardagsaktiviteter samt färre fallolyckor för äldre personer med demenssjukdom? En randomiserad studie" med Dnr 2011-205-31M. Studien förväntas leda till direkt nytta för forskningspersonen samt bidra till ett resultat som kan vara till nytta för forskningspersonen eller någon annan som lider av samma eller liknande sjukdom eller störning. Forskningen förväntas även innebära en obetydlig risk för skada och ett obetydligt obehag för forskningspersonen.

5. Forskningsetiska överväganden

5:1 Redogör för alla risker som deltagandet kan medföra

Dessa kan vara t.ex. fysisk eller psykisk skada, smärta, obehag eller integritetsintrång på kort eller lång sikt. Ange vilka åtgärder som har vidtagits för att förebygga riskerna som nämns ovan samt vilken beredskap som finns för att hantera sådana komplikationer. Ange vilka/de metoder som kommer att användas för att efterforska, registrera och rapportera oönskade händelser.

Interventionen kommer att innebära att personen med demens kommer att resa hemifrån till öppenvårdsenheten vid Geriatriskt centrum två gånger per vecka. Att passa tider, resa, byta miljö och träffa nya människor kan skapa obehag i form av till exempel oro, ångest eller förvirring hos personen med demens. Personalen i projektet har erfarenhet att arbeta med personer med nedsatt fysisk och kognitiv funktionsnedsättning och är därför uppmärksamma på om dessa obehag uppstår. Om forskningspersonen upplever obehag att åka till Geriatriskt centrum så finns möjligheter att erbjuda insatser i hemmet. Försökspersonen kan även när som helst, utan att motivera orsak, avbryta sin medverkan.

Interventionen inkluderar högintensiv funktionell träning vilket innebär en möjlig risk att drabbas till exempel av fysisk skada, smärta eller andra obehag. Klinisk erfarenhet och erfarenhet från egen och andras forskning stödjer dock att fysisk träning är genomförbart i denna grupp, eftersom inga allvarliga biverkningar är rapporterade. Enheten för geriatrik har i två tidigare forskningsprojekt (Littrand et al. Phys Ther 2006; 86:489-498. Toots et al. Accepted in Journal of American Geriatric Society) utvärderat samma träningsprogram bland personer med demens på särskilt boende. I dessa studier förekom inga allvarliga biverkningar, dessutom kunde träningen genomföras på liknande sätt (avseende närvaro, intensitet och obehag) för personer med demenssjukdom jämfört med personer utan demenssjukdom (Littrand et al. Phys Ther 2006; 86:489-498). För att minimera riskerna kommer träningen att vara individanpassad och ledas av sjukgymnaster med erfarenhet av högintensiv träning bland äldre personer med kognitiva och

fysiska funktionsnedsättningar. Deltagarens ansvariga läkare kommer också att göra en medicinsk bedömning om forskningspersonen klarar av att delta i interventionen. Vid varje träningstillfälle kommer eventuella obehag att registreras på protokoll som använts i två tidigare forskningsprojekt på enheten (FOPANU-studien och UMDEX-studien).

Datainsamlingen kan även vara krävande för deltagarna med demenssjukdom och därför kommer även testarna att uppmanas att vara uppmärksamma på tecken av trötthet eller obehag. Ifall något sådant inträffar kommer testaren att överväga att avbryta testningen och även att åter informera forskningspersonen om möjligheten att avbryta datainsamlingen.

Yngve Gustafson, professor och överläkare vid Geriatriskt centrum, har det medicinska ansvaret för denna studie vilket garanterar forskningspersonernas säkerhet och omhändertagande.

5:2 Redogör för möjlig nytta för de forskningspersoner som ingår i projektet (gäller särskilt behandlingsforskning)

Det är idag ovanligt, till skillnad från andra neurodegenerativa sjukdomar som till exempel Multipel Skleros (MS) och Parkinsons sjukdom, att landsting och kommuner erbjuder personer med demens rehabiliteringsperioder för sin grundsjukdom. Studien förväntas därför fylla ett behov hos personer med demenssjukdom inklusive närstående och leda till direkt nytta för forskningspersonerna som lottas interventionsgruppen. Interventionen förväntas ha positiva effekter på följder av demenssjukdomen som till exempel försämring i ADL och fysisk funktionsförmåga, förekomst av BPSD, social isolering, depression och närståendebelastning. Positiva effekter på dessa faktorer kan öka livskvalitén och möjliggöra att försökspersonen kan fördröja flytt till särskilt boende. Forskningspersoner som lottas kontrollgrupp kommer att genomgå olika tester och frågeformulär. De kommer att erbjudas information om testresultaten. Testresultaten kommer att gås igenom av den medicinskt ansvariga läkaren i projektet och om något allvarligt uppdagas kommer forskningspersonen att erbjudas att ansvarig läkare vid hälsocentralen eller öppenvårdsenheten vid Geriatriskt centrum informeras.
Nyttan för forskningspersonerna förväntas vara mycket större än de risker som ett deltagande skulle kunna innebära.

5:3 Identifiera och precisera om eventuella etiska problem (fördelar/nackdelar) kan uppstå i ett vidare perspektiv genom projektet (Se p. 5:3 i Vägledning till ansökan)

Här kan redovisas om exempelvis vissa grupper (andra än de forskningspersoner som ingår i forskningsprojektet) kan komma att utpekas/få hjälp som ett resultat av studien.

I Sverige finns idag cirka 160 00 personer med demenssjukdom, och inkluderat närstående och vänner påverkas sammanlagt omkring en miljon människor. Demens kan innebära ett stort lidande då det är en sjukdom som påverkar hela livssituationen för den som drabbas och dess närstående. Eftersom antalet personer med demens kommer att öka dramatiskt i framtiden förordar WHO att demens bör prioriteras som ett globalt folkhälsoproblem. De negativa konsekvenserna vid demenssjukdom är många och komplexa. Det är därför av stor vikt att utveckla effektiva metoder. Trots detta är ofta personer med kognitiv nedsättning exkluderade från studier. Det kan tyckas vara oetiskt att exkludera personer med demens från att utvärdera interventioner som är effektiva för andra patientgrupper.

Den komplexa problematiken vid demenssjukdom indikerar ett behov av personcentrerad multidimensionell interdisciplinär rehabilitering för att uppnå optimal effekt, det vill säga att återskapa eller bibehålla bästa möjliga funktionella förmåga, samt skapa förutsättningar för oberoende och deltagande i samhället. Trots detta är personcentrerad multidimensionell interdisciplinär rehabilitering mycket lite studerat bland personer med demens. Dessutom är det idag ovanligt, till skillnad från andra neurodegenerativa sjukdomar som till exempel Multipel Skleros (MS) och Parkinsons sjukdom, att landsting och kommuner erbjuder rehabiliteringsperioder specifikt för demenssjukdom. Demenssjukdomarnas symtom och progressivitet skulle kunna medföra att interventionen inte resulterar i positiva effekter. Dock har studier som studerat effekter av enskilda interventioner eller i kombination med en begränsad mängd olika åtgärder visat positiva resultat i denna patientgrupp.

6. Redovisning av resultaten

6:1 Hur garanteras forskningshuvudmannen och medverkande forskare tillgång till data (anges vid t.ex. uppdragsforskning) och vem ansvarar för databearbetning och rapportskrivning?

(Se p. 6:1 i Vägledning till ansökan)

Medverkande forskare ansvarar för databearbetning och rapportskrivning. Arbetet görs oberoende från bidragsgivare.

6:2 Hur kommer resultaten att göras offentligt tillgängliga? Kommer studien att insändas för publicering i tidskrift eller publiceras på annat sätt? (Se p. 6:2 i Vägledning till ansökan)

Ange i vilken form resultaten planeras offentliggöras samt tidsplan för detta.

Beskrivning av rehabiliteringsprogammet och resultaten från studien kommer att spridas genom vetenskapliga publikationer samt genom nationella och internationella konferenser och presentationer/föredrag för till exempel intresseföreningar. Resultaten från studien bör även kunna användas inom olika yrkeskategoriers grund- och fortutbildning.

6:3 På vilket sätt garanteras forskningspersonernas rätt till integritet när materialet offentliggörs/publiceras?

Redovisas resultat på statistisk gruppnivå? Beskriv procedurer eller metoder för avidentifiering/anonymisering.

Resultaten kommer att redovisas på gruppnivå och utan att nämna specifika hälsocentraler eller mottagningen vid Geriatriskt centrum. I datafilerna som används för analyser avidentifieras forskningspersonerna, det vill säga inga namn eller personnummer kommer att finnas registrerade.

7. Redovisning av ekonomiska förhållanden och beroendeförhållanden

Redovisning enligt punkterna 7:1-7:3 syftar till att tydliggöra alla direkta eller indirekta förhållanden, som kan tänkas påverka forskarens relation till forskningspersonerna (vid t.ex. informations-, samtyckes-, genomförandeprocedurer).

7:1 Vid uppdragsforskning

Ange uppdragsgivaren t.ex. ett företag (vid klinisk läkemedelsprövning eller prövning av andra nya produkter), en organisation eller en myndighet.

Namn:	Kontaktperson:
Adress:	Telefon/mobiltelefon:

Ange uppdragsgivarens relation till forskningshuvudmannen/medverkande forskare, t.ex. anställningsförhållande

7:2 Redovisa eventuella ekonomiska överenskommelser med uppdragsgivare eller andra finansiärer (namn, belopp)

Vid klinisk läkemedelsprövning bör hänvisning ske till ingånget avtal med sjukvårdshuvudmannen. Liknande överenskommelser kan förekomma vid annan uppdragsforskning och bör redovisas på samma sätt. Separata överenskommelser med den/de som ska genomföra forskningen ska redovisas. Belopp som kommer att erhållas för studien/ersättning till kliniken/genomföraren, vad ersättningen bör täcka och ev. belopp som erhålls per forskningsperson, bör också anges här.

Inga ekonomiska överenskommelser förekommer.

7:3 Redovisa forskningshuvudmannens, huvudansvarig forskares och medverkande forskares egna intressen

Här redovisas t.ex. aktieinnehav, anställning, konsultuppdrag i finansierande företag, eget företag som kan få (direkt eller indirekt) ekonomisk vinst av forskningen.

Ej aktuellt.

8. Undertecknande

Behörig företrädare för sökande forskningshuvudman enligt p. 1:2.

Ort: Umeå Datum:

Signatur: ______________________________

Namnförtydligande: Ann Sörlin

Tjänstetitel: Prefekt

Undertecknad forskare som genomför projektet (kontaktperson) enligt p. 1:3 intygar härmed att forskningen kommer att genomföras i enlighet med ansökan.

Ort: Umeå Datum:

Signatur: ______________________________

Namnförtydligande: Håkan Littbrand

Tjänstetitel: FoAss

9. Förteckning över bilagor (Se p. 9 i Vägledning till ansökan)

Dokument som, i tillämpliga fall, ska bifogas *om inte motsvarande information finns i blanketten* har markerats med x. Markera de bilagor som skickas in med denna ansökan.

Insänd med ansökan	Bil nr	Beskrivning	Klinisk läkemedels-prövning	Annan forskning
☐	1	Deltagande forskningshuvudmän och medverkande forskare (kontaktpersoner) vid forskning där mer än en forskningshuvudman deltar. Se p. 1:4	x	x
☒	2	För fackmän avsedd forskningsplan, vid behov även för lekmän avsedd bilaga. Se p. 2:1 och Vägledning till forskningsplan/forskningsprotokoll (program)	x	x
☐	3	Annonsmaterial för rekrytering av forskningspersoner. Se p. 3:1 och i Vägledning till ansökan p. 3:1	x	x
☒	4	Skriftlig information till dem som tillfrågas. Se p. 4:1 och Vägledning till forskningspersonsinformation och (i förekommande fall) separat samtyckesformulär	x	x
☒	5	Enkät, frågeformulär. Se p. 2:4	x	x
☐	6	Gemensam EU blankett (gäller fr.o.m. den 1 maj 2004), gäller även vid ändring. För information se Läkemedelsverkets hemsida, www.lakemedelsverket.se	x	
☐	7	Sammanfattning av protokollet på svenska	x	
☐	8	Prövarhandbok alt. bipacksedel/produktresumé/IB	x	
☒	9	Intyg från verksamhetschef/motsv. om resurser för forskningspersonernas säkerhet. Se p. 1:5 och förslag till utformning av resursintyg i Vägledning till ansökan p. 1:5	x	x
☒	10	CV för forskare (samma som p. 1:3) med huvudansvar för genomförandet, redovisa forskarens (- arnas) kompetens av relevans för studien. Se Vägledning till ansökan p. 1:3	x	x
☐	11	Beskrivning av ersättning till forskningspersoner. Se p. 3:6 och i Vägledning till ansökan p. 3:6	x	x

Övriga bilagor som bifogas ansökan:
